# Recent Advancements in Nanobiosensors: Current Trends, Challenges, Applications, and Future Scope

**DOI:** 10.3390/bios12100892

**Published:** 2022-10-18

**Authors:** Madhusudan B. Kulkarni, Narasimha H. Ayachit, Tejraj M. Aminabhavi

**Affiliations:** 1 Department of Research & Development, Renalyx Health Systems (P) Limited, Bengaluru 560004, Karnataka, India; 2 Department of Physics, Visvesvaraya Technological University (VTU), Belagavi 590018, Karnataka, India; 3 School of Advanced Sciences, KLE Technological University, Hubballi 580031, Karnataka, India

**Keywords:** nanobiosensor, nanomaterials, synthesis, nanotechnology, microfluidics, healthcare, point-of-care-testing (POCT)

## Abstract

In recent years, there has been immense advancement in the development of nanobiosensors as these are a fundamental need of the hour that act as a potential candidate integrated with point-of-care-testing for several applications, such as healthcare, the environment, energy harvesting, electronics, and the food industry. Nanomaterials have an important part in efficiently sensing bioreceptors such as cells, enzymes, and antibodies to develop biosensors with high selectivity, peculiarity, and sensibility. It is virtually impossible in science and technology to perform any application without nanomaterials. Nanomaterials are distinguished from fine particles used for numerous applications as a result of being unique in properties such as electrical, thermal, chemical, optical, mechanical, and physical. The combination of nanostructured materials and biosensors is generally known as nanobiosensor technology. These miniaturized nanobiosensors are revolutionizing the healthcare domain for sensing, monitoring, and diagnosing pathogens, viruses, and bacteria. However, the conventional approach is time-consuming, expensive, laborious, and requires sophisticated instruments with skilled operators. Further, automating and integrating is quite a challenging process. Thus, there is a considerable demand for the development of nanobiosensors that can be used along with the POCT module for testing real samples. Additionally, with the advent of nano/biotechnology and the impact on designing portable ultrasensitive devices, it can be stated that it is probably one of the most capable ways of overcoming the aforementioned problems concerning the cumulative requirement for the development of a rapid, economical, and highly sensible device for analyzing applications within biomedical diagnostics, energy harvesting, the environment, food and water, agriculture, and the pharmaceutical industry.

## 1. Introduction

In recent times, nanomaterials have gained much interest because of the need to control substances required by the human body and the environment. Usually, nanostructured materials embrace at least below 100 nm in one dimension (1-D) [1]. The production of nanomaterials with definite properties is a substantial subsection of nanotechnology. The term nanotechnology deals with materials having nanoscales from subnanometer to a few hundred nanometers in size, composed of organic matter, carbon, and metal oxides [2,3,4,5,6]. Nanotechnology relies on the ability to design, deploy, and develop, structures in the nano environment. These materials are called nanomaterials. Nanomaterials are often famed for fine to coarse particles due to their exceptional properties, like large surface-to-volume area, quantum mechanics effect, enhanced stability, and reactivity that provide a wide variety of advantages over regular materials in domains like electronics, medicine, agriculture, food, and the pharmaceutical industry [7,8,9,10,11]. Nanomaterial synthesis describes the methods used for generating nanomaterials that arise widely in nature and are the entities of investigation in numerous fields, such as physics, chemistry, biology, geology, and electronics. The nanomaterial differs in different size, dimensions, shape, and morphology [12]. In order to achieve unique properties, the controlled nanomaterial synthesis and fine-tuning parameters require a good knowledge of reaction concentration, temperature, and time optimization [13,14]. This may further lead to the advent of multifunctional and novel nanotechnologies. In the current scenario, it is virtually impossible to carry out any application without nanomaterial synthesis. Nanomaterials find application in almost all fields of science and technology. Nanomaterial synthesis is essential for sensing, testing, and diagnostic applications [15,16,17].

**Figure 1:**
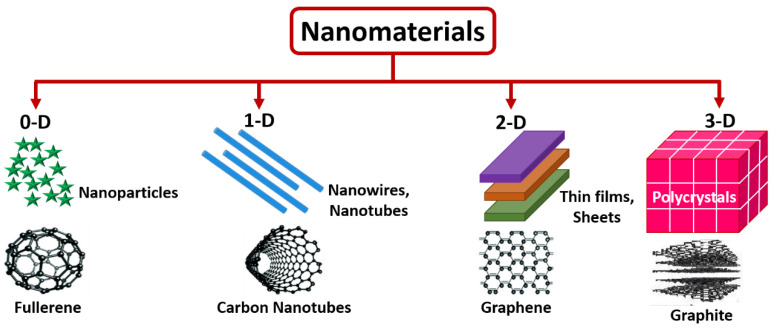
Schematic of 0-D, 1-D, 2-D, and 3-D, nanostructured materials.

Nanomaterials can be considered in either of these methods in terms of appearances wherein zero-dimension (0-D) is static at a solitary point (Ex: Fullerene), one-dimensional (1-D) can retain only a solo variable (Ex: Carbon nanotubes), two-dimensional (2-D) possesses a couple of variables (Ex: Graphene), three-dimensional (3-D) has all three variables (Ex: Graphite) [18,19]. Figure 1 shows the representation of 0-D, 1-D, 2-D, and 3-D nanomaterials. Further, nanomaterials are very important and play a dynamic role in pharmaceutical, clinical, biochemical, biological, and biomedical science, since their decisive nanosize dimensions are closer to cells, genetic molecules, microorganisms, and tissues. As in the nano environment, the physical, optical, chemical, and biological, properties are highly inclined by the particle dimension and pattern appearance. Due to their unique properties, the capability to produce nanomaterials with superb and meticulous quality is necessary for numerous application areas. The nanomaterial synthesis comprises two strategies: top-down and bottom-up, respectively. The fundamental parameters of synthesizing nanomaterial comprise precursor concentration, reaction time, and temperature. The parameters mentioned above play a considerable role in producing nanomaterials effectively and efficiently. The intelligent utilization of nanostructured materials is prophesied to increase the performance of biomolecular electronic devices with high selectivity, sensitivity, specificity, and the limit of detection [20,21].

**Figure 2:**
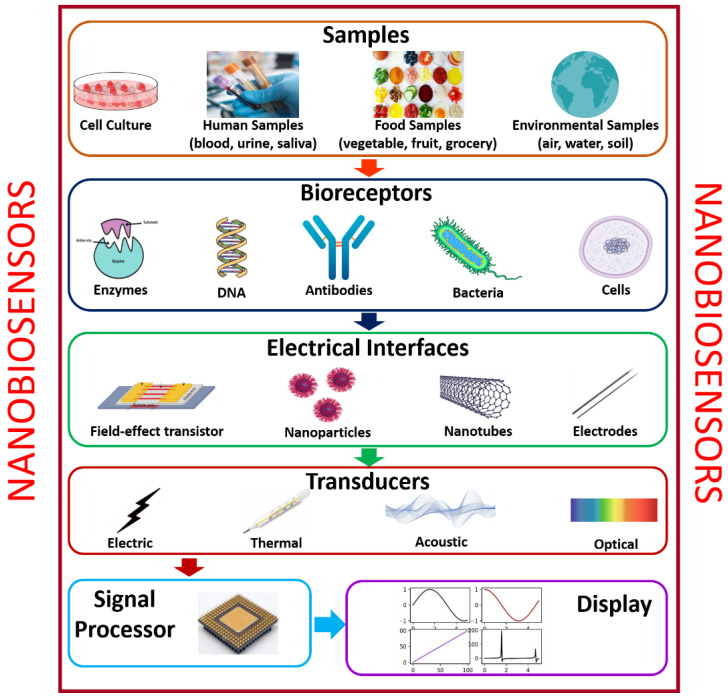
Various modules of typical biosensors.

Presently, nanomaterials are undergoing rapid progress because of their immense potential applications in catalysis, electronics, biomaterials, structural components, and biosensors. The utilization of nanomaterials such as nanotubes, nanowires, and quantum dots in biosensor medical diagnostic devices is being probed. With the development in the properties of nanomaterials in a nanoscale environment, new devices such as smart biosensors can identify the minute concentration of the desired bio-sample that are emerging faster for real-time monitoring. Herein, nanomaterials are frequently used as transducer resources that are a significant part of the development of biosensors. Biosensors are categorized into five types, namely: (i) analyte, (ii) bioreceptor, (iii) electrical interface, (iv) transducer, (v) signal processor, and (vi) display. Figure 2 shows the various modules of typical biosensors. Various analytes, like food samples, body fluids, and cell cultures, can be examined using biosensors. When the substance under test is introduced into a buffer solution, its elements are recognized (glucose, saliva, lactose, creatinine, and ammonia). Biorecognition generates a signal during the interface between the sample and components. At the same time, the transducer transmutes a biorecognition signal into a measurable electrical signal, which specifies the existence of a biochemical or biological objective. The analyte-bioreceptor interactions are proportional to the electrical or optical signals generated by the transducers, which can be linked to the smartphone via a cloud database to access and store the real-time data, which can be numerical, graphical, or tabular in nature [22].

In 1962, a biosensor was initially described by Leland Charles Clark Jr., which perceived the idea of representing its components along with the approach of incorporating a bioreceptor with a transducer device. A biosensor is an electroanalytical device, mainly used for the identification of a biochemical reaction, that syndicates a biological or electrochemical constituent with a physico-chemical indicator. Usually, a biosensor involves a biorecognition element, a transducer constituent, and an electronic device which embraces an amplifier, microprocessor, and readout. As trends in technology have progressed, the development phase of biosensors also enhanced with automation, integration, and miniaturization concepts, with advancements in microfluidic technology. Further, the biosensor reader device interfaces with the supporting electronics or signal processors that are largely in charge of the user-friendly display of the results. The broad aim of designing the biosensor is to enable rapid, expedient assay at the point of care where the real time sample was acquired.

Microfluidics is the part of science and technology that deals with the design, development, and fabrication, of miniaturized biosensor devices to regulate, correlate, or adapt micro/nano fluids ranging from 1–100 microns in dimension [23]. This emerging cutting-edge technology is rewarding rapidly in several areas, in particular with nanotechnology, microelectronics, and molecular biology. Microfluidic devices have enlarged the current state-of-the-art to harness numerous leads, such as the better handling of reagents, high reproducibility, accurately controlled size, and minuscule volume allied with nanomaterial synthesis in a microscale platform, which has the potential to be used for diverse applications. It can regulate the physical elements like particle size, shape, and morphology of the nanocarriers, which would be extremely beneficial to increase its performance. Microfluidics offers numerous potential benefits in the biochemical and biomedical domains due to their superior control over concentration, high throughput, and safer working environment. Thus, it is very much clear that these improvements in microfluidic systems associated with nanotechnology are the outset to overlay the path for a cumulative number of studies to switch, in the future, from traditional-based approaches to lab-on-a-chip platforms. Diagnostics is of clinical importance both for the detection of disease and therapeutics. Further, microfluidic biosensing provides significant research opportunities, especially for clinical diagnosis, because of its various benefits. Early diagnostics plays a vital role in detecting (prevention) or resulting (prognosis) of a disease that can be executed with a POCT platform [24,25]. POCT includes instant testing near a patient at the locality where care is to be taken to enhance the healthcare devices. One of the essential components of POCT is the biosensor with an integrated device output.

This review critically discusses two broad areas: nanomaterials and biosensors. An overview is provided with classifications and methods involved in the nanomaterial synthesis and development of biosensors with design optimization, material, fabrication, and the testing of real samples. Specific emphasis is given to applications of nanobiosensors using microfluidic technology. This also discusses the topical state-of-the-art with comparative analysis in the field mentioned above. Further, discussions are carried out with recent advancements, challenges, and limitations, associated with nanobiosensors. Finally, the future scope of the area of nanobiosensors has been discussed.

## 2. Nanomaterial Synthesis

**Figure 3:**
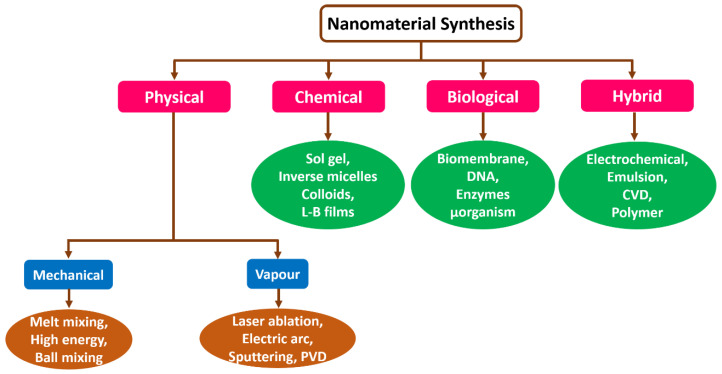
Schematic representation of several techniques that are used for the synthesis of nanomaterials.

There are several techniques to synthesize nonmaterial through different kinds of processes. Generally, a nanostructure such as nanoclusters, nanorods, nanotubes, and nanowires, can range from 1 nm to 100 nm. Nanomaterials can be produced using diverse methods that depend on approach, reagent concentration, temperature, and time. Further, these structures can be generated by fading the size or constructing molecules from micro to nanomaterial [26]. Nanomaterials and fluid particles diffuse and are synthetic by these methods. Figure 3 depicts the several techniques involved in synthesizing nanomaterials. Usually, for producing nanomaterials, physical, chemical, biological, and hybrid, processes are opted based on the application [27,28,29]. Commonly, these methods are executed and classified under the conventional approach. Here, a nanomaterial synthesis method selection relies on the material of interest or type of nanomaterials, like dimensions, sizes, and quantity [30,31].

The two main approaches to nanomaterial synthesis can be categorized as top-down and bottom-up.

### 2.1. Top-Down Approach

Herein, the Big Structure Can Be Outwardly Controlled during The Processing of Anticipated Nanostructures. A major demerit of this method is the presence of imperfections in the formed nanostructure [32,33]. Further, surface defects can impact physical properties because of their high aspect ratio. Here, nanomaterials are produced via a bulk complement that percolates out systematically step by step, resulting in the generation of fine particles. Further, this approach comprises several methods extensively used in producing the mass production of nanomaterials, like electron beam lithography, sputtering, laser ablation, nanolithography, and thermal decomposition [34].

#### 2.1.1. Nanolithography

Nanolithography is an investigation of synthesizing nanoscale with a framework of a minuscule feature generally from 2 nm to 100 nm range. Several nanolithographic procedures are accessible for producing nanomaterial [35], for instance, electron-beam, scanning probe lithography, optical, and nanoimprint. The essential advantage of this process is making a cluster from a single nanomaterial with a quantified structure and size [36,37]. Further, the only shortcoming is the need for expensive, bulky, and sophisticated instruments.

#### 2.1.2. Mechanical Milling

This is one of the extensively used methods to produce numerous nanostructured materials. It is used for post-tempering and the purifying of produced nanomaterials for high throughout production, wherein many components are powdered in an idle situation [38,39]. The operating principle in mechanical milling is a plastic distortion that drives to a nanomaterial with varied size, structures, fracture consequences in condensed nanomaterial appearance, and cold bonding consequences in improved nanomaterial dimension [40].

#### 2.1.3. Sputtering

Sputtering integrates the ousting of nanostructures on a surface morphology layer by exorcizing them from striking with ions [41,42]. Further, sputtering is often utilized for the confession of a thin film of nanostructures lagged by a mitigating procedure [43]. The layer of the film, reaction time, reagents concentration, and the thermal range of substrate form and anneal the size and structure of synthesized nanomaterials.

#### 2.1.4. Thermal Decomposition

Thermal decomposition is an endothermic biochemical corrosion method that is generally produced by heating the intrudes engaged in connection with the prepared precursor [44,45]. At the anticipated temperature range, fundamentals will chemically become decayed, while undergoing a chemical reaction creating nanomaterials.

### 2.2. Bottom-Up Approach

Herein, the Atom-Sized Elements are Shadowed by Self-Assembly Resulting in the Formation of Nanomaterials [46]. During this process, the fundamental unit of a more prominent factor is the self-possessed nanomaterials. This approach has been utilized for producing quantum dots (QDs) during epitaxial expansion and the generation of nanostructures from colloidal distribution. Usually, this method produces fewer flaws and more homogenous chemical configurations [47]. Typically, this method is the accretion of material from slighter particles to certain clusters of nanostructures. Furthermore, this method includes several methods like sol-gel, hydrothermal, chemical vapor deposition (CVD), spinning, and pyrolysis [48].

#### 2.2.1. Hydrothermal

The hydrothermal method has been valued and has acquired attention from several scientists and researchers in the generation of nanomaterials [49]. The hydrothermal-based nanomaterial synthesis has been effective for executing authoritative entities such as chemical oxides for sensing, microporous quartzes, multi-layered ceramic oxide, and hypersonic electrodes [50]. Hydrothermal deliberates the nanomaterial synthesis via biochemical solutions operated beyond the steaming point of water in a vacuum-packed temperature reaction above the atmospheric pressure [51]. This method can be simple and precise for obtaining the desired nanomaterials. However, it needs classy autoclaves and lacks safety parameters during the process.

#### 2.2.2. Sol-Gel

Sol-gel is one of the most preferred methods to synthesize nanomaterial due to its simple process. In this, a colloidal absorption of solid is deferred in a fluid stage, and gel is a solid macroparticle submerged in a chemical reaction [52]. Usually, this method defines a wet-chemical procedure, including a biochemical reaction accomplishing as a precursor for a united structure of diverse materials. Here, chlorides and metal oxides prepare the precursor in the sol-gel method [53]. The chemical reaction is later dispersed in an analyte using a magnetic stirrer, sonication, or shaking. The following chemical reaction analyte includes both solid and liquid phases [54]. Further, the acquired reaction is unglued, and the filtered reaction is improved by sedimentation, filtration, and centrifugation, further eradicated by drying it in the micro hot air oven.

#### 2.2.3. Chemical Vapor Deposition (CVD)

The CVD method bonds particles on a thin film substrate using gaseous reagents [55]. The solution chamber is the intermediate for conducting deposition at room temperature gradient by an amalgamation of gas atoms. Here, a reaction occurs when a warm material originates in contact with combined gas [56]. This process produces a thin film substrate on the surface, which is enhanced and employed based on the application. CVD has benefits such as being highly pure, uniform, and has strong nanomaterials [57]. Here, the limitation is the need for sophisticated instruments and the release of gaseous products, which can be highly toxic.

#### 2.2.4. Pyrolysis

Pyrolysis is mainly used in industries for producing very large-scale nanomaterials. It includes burning a reaction with a blaze at a high steam rate [58,59]. The precursor can be either liquid or vapor converted into an oven at a very high-pressure rate through a small hole where the flame can be functional [60]. This formed combustion is later air, characterized to recover the nanomaterials. The advantages of pyrolysis are easy to use, are economical, efficient, and are effective, with significant consequences.

**Figure 4:**
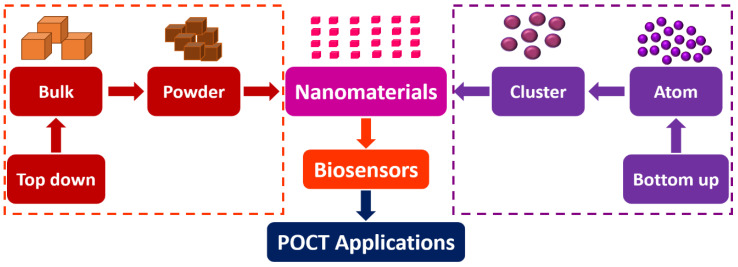
Schematic of the top-down and bottom-up approaches of nanomaterials synthesis.

Figure 4 shows a schematic of the top-down and bottom-up approaches of nanomaterials synthesis. Table 1 summarizes the different methods of nanomaterials synthesis.

Further, producing nanomaterial in the microfluidic device is one of the most widely used techniques in recent times as it finds application in biochemical and biomedical domains due to its extensive benefits, such as low cost, simple, rapid, transportable, transparent operation, micro/nanoscale process, manipulability, and high-throughput during the synthesis of nanomaterial. It also plays a dynamic role in creating exact nanostructures with varied size and morphology of the materials. Here, microfluidic technology eradicates the requirement of sophisticated instruments and is well suited for point-of-care applications because of its automation, integration, and miniaturization (AIM) concepts [65]. Being time-consuming, the necessity of expensive autoclaves, batch reactors, and high-power consumption are just a few factors associated with the current conventional techniques. Further, microfluidic deals with two kinds of methods in controlling fluid flow: chamber-based and continuous-flow-based strategies [66,67]. In the chamber-based process, the fluid will be statically placed inside the microreactor over some time. On the other hand, in the continuous-flow-based approach, the fluid will pass along the capillary concerned with time. Table 2 summarizes the comparison of traditional and microfluidic platforms for nanomaterial synthesis.

## 3. An Overview of Nanobiosensors

Nanobiosensors basically emerged from two areas: nanotechnology and biosensors. Typically, these are the sensors that are developed from nanomaterials and, fascinatingly, these are not the dedicated sensors that can identify the nano range measures and activities. Nanomaterials are of exclusive ability to humanity from nanotechnology with vital physiochemical properties that are extremely diverse from the same nanostructures produced at a bulk scale. Here, nanomaterials play an effective role in the detecting mechanism of biosensing technology. Further, integrated devices of nanomaterials with electrical components lead to microelectromechanical systems (MEMS) that are responsible for the means of transducers [68,69].

In the recent past, many nanomaterials have been explored in the tool of electrical and mechanical properties for their utilization in enhanced biological signal processing. A few extensively employed nanomaterials in sensing applications include nanowires, nanotubes, nanorods, and thin films, composed of crystalline matter. These can range from using amperometric devices for enzyme-based glucose sensing to using quantum dots (QDs) as fluorescent agents for binding detection and even bioconjugated nanomaterials for targeted biomolecular detection. These include colloidal nanomaterials that can be employed for immunosensing and immunolabeling applications by coupling with antibodies. These substances can also be utilized to improve detections made using an electron microscope. Additionally, metal-based nanomaterials are particularly good for electronic and optical applications. By taking advantage of their optoelectronic characteristics, they can be effectively used to detect nucleic acid sequences. Several nanomaterials have been described to investigate their properties and topical applications in biosensors. The attributes of the biosensor for better efficiency includes response time, selectivity, sensitivity, and linearity [70,71].

Research studies show a persistent upsurge concerning many nanomaterials with the scope of interest to implement either transducer or receptor operation to improve their multi-detection sensitivity and capability. Further, nanoparticles, nanotubes, quantum dots, and other biological nanomaterials, are examples of these nanomaterials [72,73]. These may support the transducer, bio-recognition component, or both. To enable the quick examination of numerous chemicals in vivo, nanosensors, nanoprobes, and other nanodevices, have revolutionized biochemical and biological investigation disciplines. A wide range of nanomaterials with various characteristics, including small size, faster, shorter distances for electrons to traverse, reduced power, and lower voltages, have emerged in recent years [74,75]. Nanomaterials like metal nanoparticles, oxide nanoparticles, magnetic nanomaterials, carbon materials, and quantum dots, are now used as an outcome of significant advancements in the field of nanotechnology to enhance the electrochemical signals of biocatalytic measures that take place at the electrode and electrolyte interference. Functional nanomaterials attached to biological molecules such as proteins, peptides, and DNA, have been produced for use in biosensors. The top-to-bottom method entails the use of chemical (isotropic) and physical (anisotropic) processes to micro/nano machine macroscopic materials down to the necessary nanoscale environment [76]. This process comprises a mixture of methods like laser ablation, lithography, chemical etching, and ion milling. On the other hand, in a bottom-up strategy, the production of an initial critical mass is followed by the accretion of material, which builds the material. Further, molecular beam epitaxy, evaporation, physical or chemical vapor deposition, and protein-polymer nanocomposites, are used under bottom-up nanofabrication [77,78].

### 3.1. Evolution of Nanobiosensors

IUPAC describes the nanobiosensor as an independent integrated device capable of using a biological technique to offer a precise quantitative or semi-quantitative analytical readout that a recognition element is physically reaching a transducer element [79]. The nanobiosensors have shown potential interest and recompenses to harness biological samples for the early detection of viruses and pathogens after the discovery of nanomaterials. There are mainly three generations of nanobiosensors [80]. In 1962, the electrode proposed by Clark [81] is known as the first generation of nanobiosensor, and this is based on oxygen dependence. In our earlier work, we have reported in detail about the three generations of nanobiosensor [22]. Table 3 summarizes the evolution pathway of nanomaterials and biosensors that have undergone different development phases over the years.

After several improvements, the nanobiosensor has created an enormous trust toward balancing technology and healthcare, especially in biomedical applications where it is a boon for the early detection of several diseases that can help physicians make further decisions based on the initial test results [105]. Additionally, nanobiosensors are already employed as long-term commercial products and can be produced for specific uses.

### 3.2. Development of Nanobiosensors

Nanobiosensors are non-intrusive, responsive, and are designed using novel nano and biotechnology approaches. The real-time sensitive indicators generated by these sensors can be readily gathered and investigated. The nanobiosensor is encompassed of three modules: genetic probe comprising materials based on affinities like enzyme–substrate, antibody–antigen, DNA, and cell-based interactions. Further, a transducer translates biological data into an electrical signal and a data footage unit that includes loading and transmitting the information. Biological components can be nucleic acid, antibodies, and enzymes. Bioanalytes can be sensed by the biological probes linked with several synthesized nanomaterials like magnetic, metallic, graphene oxide, quantum dots, and CNTs. Transducers using electrochemical signals are potentiometric, amperometric, and voltammetric signals comprising fluorescence, colorimetric, and optical fibers. Different kinds of advanced nanomaterial have been employed for the production of novel nanobiosensors [106,107,108]. Thus, various forms of nanomaterials, from classy to economical, have been incorporated for designing and developing robust nanobiosensors.

The development of the nanobiosensor for the detection of biological, biomaterial, and electrochemical products, such as cholesterol, choline, dopamine, vitamin, blood glucose, creatinine, albumin, drugs, enzymes, protein, and nucleic acid, includes the conversion of biological or chemical reactions to electrical output [109]. This specifies the composition or concentration, rheological properties, amplitude, energy, pH, polarization, and decay time, based on stability and sensitivity within a few seconds of time. Due to their particular diverse behaviors, gold, silver, diamond, and platinum, nanoparticles are employed in the design and development of nanobiosensors. The transducers may be definite articles, plastics, fibers, metals, ceramics, silicon, and glasses, that convert medium into determinate signals like piezoelectric, electrochemical, optoelectronic, and thermal [110,111]. Further, the optimization constraints comprise the dynamic range of concentration and sensitivity, volume of the reaction sample, sensory nanomaterials, temperature, pH, detection time, and rheological factors [112].

**Figure 5:**
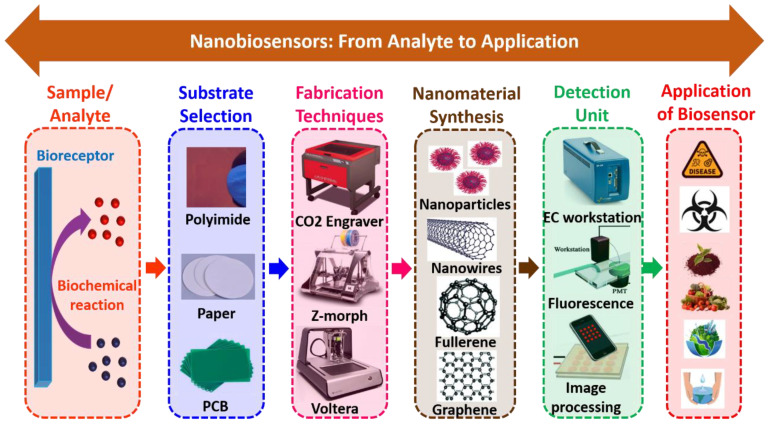
Schematic representation of nanobiosensors, from analyte to application.

Figure 5 shows the schematic representation of nanobiosensors, from analyte to application. Herein, it involves the design, development, optimization, characterization, material selection, fabrication, and testing of analytes on the miniaturized platform. Nanobiosensors are clustered into different types with prodigious contemplations on the nanostructured materials and biosensing process. These nanoparticles have great qualities that improve the sensing system. Basically, they comprise carbon nanotubes, nanowires, nanoparticles, and quantum dots (QDs) based nanobiosensors. Table 4 summarizes different types of nanomaterials used to create nanobiosensors.

Further, the production of nanobiosensors needs a critical selection of substrate for fabrication purposes, as the applied analyte on the nanomaterial surface on the biosensor should react efficiently and effectively. The materials for nanobiosensors employed must be suitable with appropriate properties. Initially, materials used for nanobiosensor fabrication were mainly silicon and glass. However, as technological advancements emerged, novel materials, including polymers, polyimide, paper, and Al foil, were used to develop nanobiosensors. Materials can be categorized into three types: polymeric, inorganic, and paper. The materials should have decent electrical, optical, thermal, and mechanical conductivity, with a high melting rate. Therefore, material selection is the key factor for manufacturing nanobiosensors, which is indispensable in POCT applications. The progressive techniques typically emphasize the principle and application of the sensing devices comprising laser ablation, 3D printing technology, and lithographical technique for several tenacities in developing nanobiosensors [119]. Further, these techniques may be attributed to large area devices, energy utilization, high resolution, impact on reaction sample, temperature parameters, crystallization, damage to the substrate, and carrier flexibility competence. The traditional method uses non-impact printing and screen printing technologies that are non-uniform, and indecisiveness, which functions on a descent-on-demand feature [120]. Table 5 illustrates various materials used for the development of nanobiosensors. Table 6 summarizes the several nanofabrication methods used in the development of nanobiosensors.

## 4. Applications of Nanobiosensors

**Figure 6:**
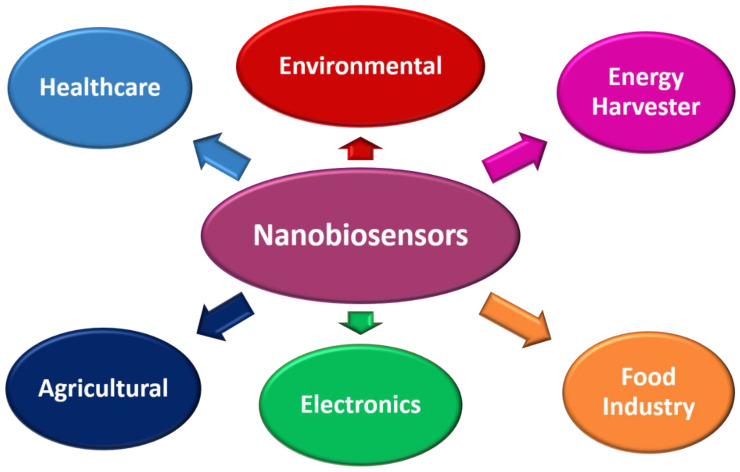
Schematic of several applications of nanobiosensors.

Nanomaterials can benefit numerous fields of study, ranging from biochemical, biomedical, pharmaceutical, agricultural, environmental, electronics, energy harvesting, and food technology [138,139,140,141]. It finds application in almost all fields of science and technology. Further, with biosensors introduction, the craze for nanomaterials has increased drastically, especially for POC applications. Since then, nanomaterials and biosensors are often called nanobiosensors. These nanobiosensors are highly versatile and multifunctional for monitoring and detecting analytes. Figure 6 shows a schematic of several applications of nanobiosensors.

### 4.1. Biomedical and Diagnostic Applications

**Figure 7:**
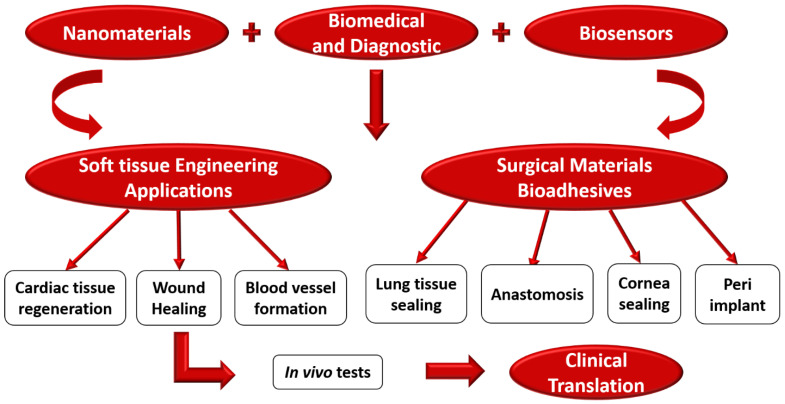
Shows the process of nanobiosensors used for various biomedical and diagnostics applications.

Since the advent of nanobiosensors, they have been used extensively for the biological identification of serum carcinogens, antigens, and the causative organisms of several metabolic ailments. The employment of nanobiosensors in detecting ailments like diabetes, allergic reactions, cancer, and other diseases based on serum analysis, best suits routine diagnostic applications. From a clinical perspective, most of the investigated and compelling benefits of nanobiosensors have several therapeutic applications that are primarily made possible for POCT. Figure 6 shows the schematic process of nanobiosensors used for various biomedical and diagnostics applications. The applications include detecting urinary tract infections, cardiovascular disease (CVD), tissue regeneration, glucose in diabetic patients, and HIV-AIDS. Further, with the initiation of the nanobiosensor there has been rapid development in the diagnosis of cancer, chronic kidney disease (CKD), and tuberculosis [142,143,144]. Further, with the inclusion of a nanoscale environment, this has been promoted and made more accurate. Identifying enzymes has been immobilized due to the incorporation of nanoparticles, which has permitted the reuse and recycling of expensive enzymes. Applying nanotechnology like NEMS and MEMS has improved the overall testing process. Microarray and biochip-based technology have made it even more comfortable, reliable, and faster to test numerous diseases. Besides, nanobiosensors have enhanced accuracies and sensitivities, making them good potential candidates for biomedical and diagnostic applications [145]. Figure 7 shows the process of nanobiosensors used for various biomedical and diagnostics applications. 

Nanobiosensors are versatile devices for biomedical screening for the early detection of several pathogens. Table 7 shows the different types of nanobiosensors in biomedical applications.

**Figure 8:**
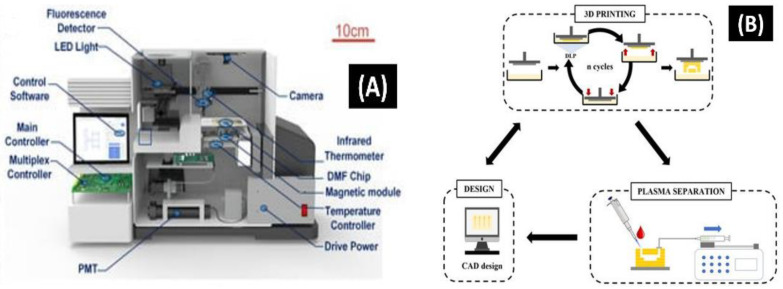
(**A**) Structure diagram of an all-in-one system for sensing nucleic acid using LAMP [156]. (**B**) The 3-D-printed microfluidic analytical devices are applied for plasma separation and the iterative fabrication process workflow [157].

Siyi Hu et al. [156] developed an automatic all-in-one digital microfluidic-based analytical device for loop-mediated isothermal amplification (LAMP) based DNA extraction and amplification, using a nano-magnetic bead with a custom-made signal processing device for real-time monitoring and detection. Herein, the study reveals that the on-chip test’s extraction efficiency was on par with that of traditional off-chip techniques. The autonomous on-chip workflow had a processing time of only 23 min, which was quicker than the traditional method with 28 min 45 s. Concurrently, the number of samples used for the analysis on-site was expressively minuscule than that used in off-site trials; only 5 µL of *E. coli* analyte was essential for DNA extraction, and 1 µL of the template DNA was used for the quantification solution mixture (Figure 8A). Further, SARS-CoV-2 DNA reference constituents were used for detection purposes, with a detection limit of 10 copies/µL. The projected system offered a POCT with a robust on-site sample to answer within 60 min.

Sandra Garcia–Rey et al. [157] demonstrated a 3-D-printed microfluidic system for blood plasma separation. Herein, the proposed idea works on one-step fabrication processes through a layer-by-layer approach. Blood is the gold-standard biological fluid for a clinical study. A technique for the rapid prototype optimization of an operational plasma separation modular device was developed using a specially formulated resin and a high-resolution 3-D printing technique (Figure 8B). In this instance, the utilization of 3-D printing exemplifies the significant impact this microfluidic technology will have on the market for plasma separation biomedical devices.

### 4.2. Environmental Applications

This is a comparatively wider area of application for nanobiosensors. This is because the environment experiences several quick changes almost every minute. Thus, it is crucial to determine toxic intermediate waste streams, that include heavy metals and air pollution. Additionally, monitoring atmospheric conditions like estimating temperature, humidity, and over-forecast features, are extremely meticulous and comprehensive tasks [158,159,160]. The nanobiosensors can be versatile in several forms of determining and monitoring. Utilizing microdevices like cantilever-based electronic probes that need minimum reaction samples are good technology invaders. Using a substrate-specific mechanism, nanobiosensors have been projected to detect biological oxygen, nitrates, and phosphates. These applications can be integrated on a single platform that allows multiplexing to sense the different contaminants, reducing the energy and time in nature. Further, these nanobiosensors are environmentally friendly and easy to use with remote access. Trends in nanobiosensor development for environmental and agricultural fields have seen progress ranging from particular sensing of mycotoxins, viruses, and signaling particles to reducing the overall time for detection. Table 8 shows the different types of nanobiosensors in environmental applications.

### 4.3. Food Industry

Nanobiosensors can also optimize numerous other detections, such as in the chemical and food industries. In food industries, substrates mixes into biological reactors, feeding nutrient stuff for different applications that can be controlled using these nanobiosensors. On an industrial scale, several commercial ingredients’ separation and preparation can be improved with these nanobiosensors [170,171,172]. For instance, a metallurgical process requires the separation of impurities that are present in a complex form and are gathered in the form of ores. Here, nanobiosensors can be employed to selectively separate the contaminants by experimenting with different alignments of detecting enzymes. Using highly sophisticated and precise molecules, mainly endocrine-disrupting substances, carcinogens, and unstable intermediates causing the disruption of appropriate hormonal systems in living beings have been discovered. Emerging biological and biochemical tests, combined with bioengineering-based revolutions, are certainly accessible applications of these detecting nanobiosensors. In the food industry, a few pollutants can be detected using nanobiosensors during harvest, storage, and transportation. Table 9 shows the different types of nanobiosensors in the food industry.

### 4.4. Electronics Applications

Generally, microelectromechanical systems and electronic circuits have been constructed using silicon wafers. Integrated circuit commercialization started in 1965 using silicon-processing technologies in the microelectronics sector. Over time, there has been an increase in the progress made in shrinking circuit size [190]. Moore Law’s 1965 prediction sped up the development of smaller integrated circuits by reducing transistor size, increasing transistor density, and improving operating frequencies. Field effect transistors were first sized below 100 nm in 2000, ushering in the silicon nanoelectronics era. The production of chips using nanostructured materials has been considered by many businesses, including iMac G5, IBM, Intel, etc. In this context, graphene, mxene, CNTs, quantum dots, and fullerene can be discovered for the electronics nanosensor manufacturing industries. For instance, nantero has created the NRAM (nanotube-based/non-volatile random access memory) chip employing CNTs that may function as dynamic memory cells [191]. In 2006, IBM produced transistors based on CNTs [192]. Other nanomaterials are being studied for the manufacture of actuators, transducers, sensors, and high-k dielectrics, which include ferroelectric oxides barium titanate (BaTiO_3_), which lead barium-strontium titanate ((Ba-Sr)-TiO_3_) and zirconate titanate (Pb(Zr-Ti)O_3_). Further, a technique for building electronic circuits by mounting electrical devices on flexible plastic substrates is known as flexible electronics, commonly referred to as flex circuits. These flexible electronics are widely used as wearable nanobiosensor devices for sensing various biological parameters, such as glucose, sweat, and pulse [193,194].

## 5. Limitations, Challenges, and Current Trends of Nanobiosensors

According to the statistics, the worldwide population is predicted to reach 8.5 billion by 2030, with gradual growth expected to reach the 9.8 billion mark by 2050 [195]. This may impact the healthcare domain with further challenges in requiring more diagnosis machines for each patient, lack of testing facilities, and increased time because of huge demand. Moreover, the diagnostic costs may increase two to three times, burdening the ordinary person in a developing country like India. Thus, there is a need to develop an in-house portable, low-cost POCT device that can produce instant results with advanced incorporated technology [196,197].

Engineered nanomaterials generally provide higher electrical, thermal, mechanical, and optical properties, have nanoscale size, intensify anticipated signals, and are well-suited to biological particles [198]. For instance, carbon materials can be used to conjugate biomolecules such as an antibody, enzymes, cells, and nucleic acids. Several researchers have reported that the inclusion of nanomaterials may drastically improve the performance of biosensors, selectivity, specificity, and lower detection limit by several orders of magnitude. Nanomaterials demonstrate augmented chemical activity, surface-to-volume ratio, mechanical potency, improved diffusivity, and electrocatalytic properties [199]. Further, these are predicted to play a substantial role in the high enactment of biosensors with the potential to be used for various applications to probe biomolecules like viruses, pathogens, bacteria, and nucleic acids.

The field of nanotechnology has grown by leaps and bounds where several intriguing nanomaterials are extensively used for diagnostics applications with fabricated or modified biosensors on different material substrates, few nanomaterials including carbon nanotubes, graphene, quantum dots, and nanocomposites. The first major application of the developed nanobiosensor was for sensing glucose [200]. However, the commercialization of nanobiosensors as a product has faced significant challenges in the post-hype of field. The growing public and regulatory concerns about the nonexistence of international guidelines for evaluating the safety of nanobiosensors with industrial/healthcare ethical needs are the most serious issues to be addressed before these products become commercially feasible.

Along with this, the significant challenge is portraying the immobilization process that can be used to closely connect two biomolecules onto a nanobiosensor [201]. Therefore, the method utilized to immobilize a given enzyme is one of the significant aspects of fabricating a consistent biosensor. An attractive candidate for immobilizing biomolecules on a transducer superficial that effectively maintains the bioactivity of the biomolecules is a nanomatrix. The biosensors automation, integration, and miniaturization based on their properties, size, and nanostructures, are a few ongoing challenges.

In recent years, the demand for POCT has increased drastically because of its rapid, reliable, robust, and economic features for the detection, testing, monitoring, and diagnosis of biomedical samples such as blood, urine, saliva, and DNA. This demand subsists in the areas of foodborne pathogens and bacteria detection, environmental pollutant monitoring, and biochemical assay. Due to the advancement in current trends of technology incorporated with cyber-physical systems and artificial intelligence, the development of intelligent nanobiosensors has immensely increased in the commercial market [202,203,204]. Generally, the nanobiosensors area is multidisciplinary and involves science, electronics, and mechanical challenges. The next-generation nanobiosensor platforms need substantial developments in sensitivity and selectivity to address the requirements in various fields containing pharmaceutical, drug delivery, pathogen detection, and in vitro biomedical diagnostics [205,206,207].

Further, diagnostic technology development has been crucial to the advancement of medicine. The capability to detect viruses and pathogens by sensing connected nucleic acid sequences, proteins, cell receptors, organelles, enzymes, and several other biomarkers, can offer biomedical healthcare professionals, scientists, and researchers, a clear idea of patients conditions and disease pathways can be provided by the nanobiosensors [208,209]. However, several of the traditional assays presently accessible are time-consuming, need a macro capacity of analytes, and may lead to unpredicted false results. Thus, there is a huge requirement for rapid, inexpensive, trustworthy, multiplexed screening to sense a broad range of analytes. The research should focus on nanomaterials and biosensors that aim to integrate nanoelectronics, sensors, and materials, into economical, effective, and environmentally friendly nanobiosensors, with interest in numerous fields such as food analysis, diagnostics, environment, and other industries for detecting and monitoring.

**Figure 9:**
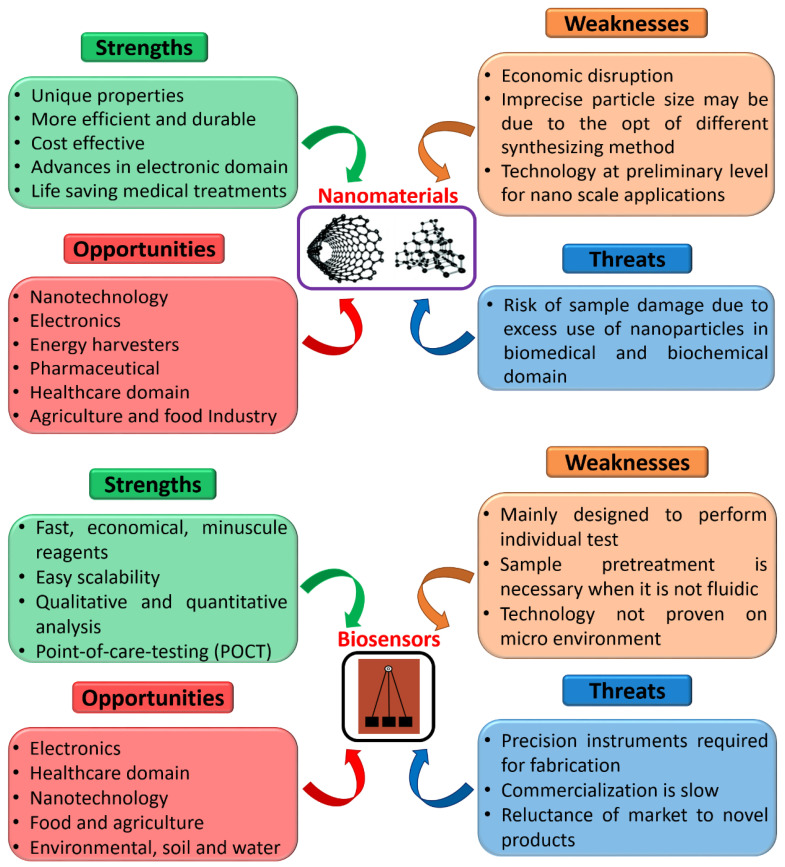
Detailed SWOT analysis of nanomaterials and biosensors.

Further, SWOT analysis (strengths, weaknesses, opportunities, threats) was executed to visualize better and understand nanomaterials and biosensors used for several applications, as shown in Figure 9. The nanobiosensors domain has exceptional strengths and limitless chances in various fields. Moreover, it is apparent that there are large areas that can be improved in respective environments with advanced technology. Considering the advantages of this domain unification, we trust that these weaknesses can be overcome in the future. Furthermore, we believe that the association of nanomaterials and nanosensors with the progression of technology will shortly pave the way for attaining smart nanobiosensors with the incorporation of artificial intelligence, machine learning, and cyber-physical systems for POCT applications in the area time scenario [210].

## 6. Conclusions and Future Scope

**Figure 10:**
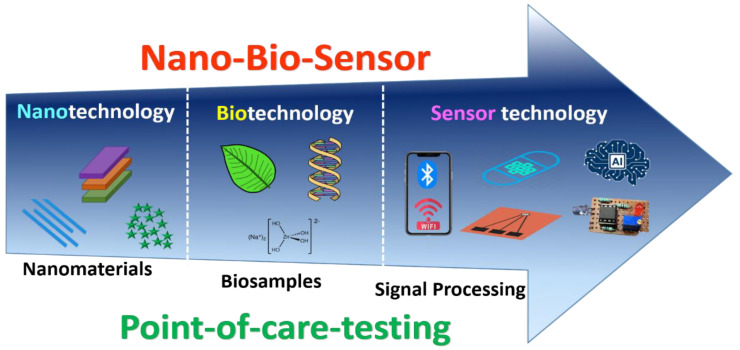
Future scope of nanobiosensors.

Nanobiosensor research aims to develop innovative technologies that can substantially contribute to the biomedical, biochemical, environmental, agricultural, and food industries, for detection and monitoring purposes. Moreover, the focus of the nanobiosensor is to benefit humanity and society. Due to the development of nanotechnology, new horizons for nanobiosensors to develop with dimensions suited for intracellular use that is submicron-sized as per the application. Attention should be given to investigating various special effects, like dimension, quantum size, and surface effect, that are exclusive to nanostructured materials synthesis and are essentially their most attractive feature. Further, the invention of novel nanomaterials is essential to be discovered to showcase even better properties for biosensing applications. Ideally, nanotechnology-based biosensors should be fully integrated within miniaturized microfluidic devices with on-chip sample handling, electronics, controller, and analysis. This will remarkably boost their operation by offering devices that are simple, portable, economic, eco-friendly, disposable, highly versatile, and diagnostic instruments. Although an extensive range of biosensors has been designed in a previous couple of decades, the futuristic goal of high throughput, inexpensive, multiplexed operation for clinical diagnostic on a microfluidic-based lab-on-a-chip (LOC) devices are yet to be accurately realized. Literally, it is still not clear which nanobiosensor designs are best suited to which diagnostic tasks. Moreover, nanobiosensors that are operational in the traditional laboratory may not be used in the clinic or field for many reasons. Well-planned multidisciplinary research should be executed that includes engineers, bioscience researchers, scientists, and doctors, to reveal more advanced and inexpensive nanobiosensors. The future scope of the nanobiosensor should be highly significant, with a slogan for automation, integration, and miniaturization. By integrating and executing advanced technological features like internet-of-things (IoT), deep learning (DL), cloud computing, data analysis, cyber-physical systems (CPS), and artificial intelligence (AI) can lead to the commercialization of the product. Figure 10 shows the future scope of nanobiosensors. It mainly includes three technologies: nano, bio, and sensor; merging altogether produces a nanobiosensor for POCT applications.

## Figures and Tables

**Table 1:** Summarizes different methods of nanomaterials synthesis.

Techniques	Reaction Temperature and Time	Morphology	Size (nm)	Advantages	Disadvantages	Ref
Hydrothermal	>220 °C and ~12–24 h	Spherical	10–200	Control over a minimal size range and suitable form	Peripheral product, high pressure required	[61]
Sol-gel	Ambient environments, ~2–4 h	Spherical	400–650	Hybrid nanoparticles are made possible by producing atoms with the exact shape and length	Contains components of the sol-gel matrix on their surfaces	[62]
Pyrolysis	5–50 °C, a few h	Sphere with a smaller diameter	1200	High pace of output	Formation of large collections	[63]
Thermal decomposition	100–300 °C, ~24–36 h	Spherical with flakes	550–600	High scalability, decent shape control, and minimal size distribution	High temperature and inert environment requirements	[64]

**Table 2:** Comparison of traditional and microfluidic platforms employed for synthesizing nanomaterials.

Variables	Conventional	Microfluidic
Device footprint	Sophisticated	Miniaturized
Reagent volume	Millilitres	Micro to nanolitres
Temperature	High	Low
Operation	Macroscale	Micro/nanoscale
Control Medium	Manual	Automatic
Execution time	More	Less

**Table 3:** Evolution of the development of nanobiosensors.

Year	Nanobiosensor Development Phase
1857	Michael Faraday was the first to report the synthesis of gold nanostructured materials [82].
1940	Fumed-based silica nanomaterials synthesized in USA [83].
1956	Lyons from the cincinnati paediatrics clinic demonstrated the first glucose enzyme electrode-based nanobiosensor [84].
1959	Richard Feynman, identified the bottom-up approach [85].
1960	Metallic nanopowder was produced for magnetic recording tapes [86].
1962	Radioimmunoassay was created by Solomon Aaron Berson [87].
1965	The direct potentiometric measurement of urea following urease hydrolysis was one of the first publications in the field of biosensors that S. Katz presented [88].
1972	For the first time, Betso et al. demonstrated that cytochrome directly transfers electrons [89].
1973	W. Mindt developed a lactate electrode [90].
1974	Norio Taniguchi, a Japanese scientist, proposed Thin-film deposition and ion beam milling and used the term “nanotechnology” for the first time [91].
1976	Granqvist and Buhrman synthesized nanocrystals by inert gas evaporation [92].
1978	First microbe-based nanobiosensors [93].
1981	Eric Drexler proposed molecular nanotechnology [94].
1983	First surface plasmon resonance immune nanobiosensors [95].
1984	Cass was the first to propose ferrocene-mediated amperometric glucose nanobiosensor [96].
1985	Discovery of fullerenes and synthesis of CNTs used as nanobiosensor [97].
1990	Pharmacia Biacore projected an SPR-based nanobiosensor [98].
1992	Bartlett et al. presented mediator-modified enzymes [99].
1995	i-STAT demonstrated a portable blood nanobiosensor [100].
1998	IUPAC introduced chemosensor in an analogy of nanobiosensor [101].
2005	Electrodeposition paints were first used as immobilization matrix for biosensors by Schuhmann [102].
2017	Girbi developed a neuron-on-chip (NoC) nanobiosensor to quantity the nerve impulse conduction [103].
2021	Kulkarni et al. introduced an aluminium (Al) foil-based nanobiosensor [104].

**Table 4:** Different types of nanomaterials are used to create nanobiosensors.

Nanomaterials	Advantages	Disadvantages	Ref
Carbon nanotubes (CNTs)	Imparts physical and mechanical properties to enhance conductivity and resistance to temperature Better surface for preventing biological component movement	Less solubility in an aqueous environment Lack of selectivity	[113]
Quantum dots	Confinement of charge carriers Size tunable bandwidth energy Excellent fluorescence	High toxicity using for in vitro systems Blinking effect	[114]
Nanowires	Good charge conduction Highly versatile Good electrical and sensing properties	No fluorescence Large amount of surfactants	[115]
Nanoparticles	High loading efficiency Good catalytic properties High immobile efficiency	Biocompatibility Average optical signal	[116]
Nanorods	Size tunable energy regulation Coupled with MEMS Plasmonic material	Metabolism varies with different materials Few in vivo studies	[117]
Metal oxide nanomaterials	Better electrical conductivity UV absorption Antimicrobial Ability of photocatalytic	Limited transfection efficiency	[118]

**Table 5:** Various substances used for the development of nanobiosensors.

Substances/Ref	Melting Point (°C)	Thermal Conductivity (W/mK)	Advantages	Disadvantages
Polymethylmethacrylate [121]	125 °C	0.16–0.18	Exceptional limpidity High toughness and mechanical strength High firmness Excellent thermal consistency Low water adsorption	Brittle Low resistance Low chemical resistance Chance of stress hitches Needs extra equipment to fabricate
Polycarbonate [122]	265 °C	0.21–0.25	Extreme impact resistance Greater clarity	Grazes easily Expensive
Polytetrafluorethylene [123]	330 °C	0.28	High-temperature tolerance Outstanding lubricity	Very smooth
Polyimide [124]	265 °C	0.12–0.36	Superior temperature adaptability High chemical resistance Extraordinary mechanical capability	High-cost fabrication
Glass [125]	1350 °C	0.86	Inexpensive Good protection power Exceptional pellucidity	Brittle Bulky
Paper [126]	210 °C	0.04	Economical Simple Automatic fluid flow Ecological	Low resolution Restricted to simple designs
Cloth [127]	120 °C	0.083–0.18	Abundant Easy-to-use Flexible	Non-biodegradable More weight

**Table 6:** Summaries of the several nanofabrication methods used in the development of nanobiosensors.

Nanofabrication	Specifications	Merits	Demerits	Ref
Lithography	Trace width = 0.05 mm	Excellent resolution	Requires clean room facility	[128]
UV-DLW	Wavelength = 405 nm	Superior resolution	Expensive tool	[129]
Voltera Inkjet printer	Trace width = 0.15 mm	Flexible substrates	Refilling of Ink such as carbon, graphene, silver	[130]
Screen printer	Trace width = 0.3 mm	Inexpensive	Less sensitive	[131]
Wax printer	100 to 500 μm	Reduce production time.	Limited materials	[132]
3D printer (FDM/SLA)	Resolution = 35–100 microns	Greater resolution and precision	Requires post-processing responsibilities like cleaning with IPA	[133]
Laser Ablation (CO_2_)	Wavelength = 10.6 µm	Precise dissection, good efficacy	Bulky and costly	[134]
Photolithography	Max width = 325 mm, maximum substrate thickness = 3 mm	Photosensitive polymers are essential	Expensive mask	[135]
Softlithography	Silicone elastomer (PDMS)	Apparent	Low thermal conductivity	[136]
Computer Numerical Control (CNC) Milling	Fast cutting speed = 8000 mm/min	Rigid design, Require less maintenance	Expensive	[137]

**Table 7:** Different types of nanobiosensors in biomedical applications.

Nanobiosensor	Nanomaterials Used	Type of Sensor	Applications (Detection)	Limit of Detection (LOD)	Ref
Antibiotic residue sensor	Au, Pt and SiO_2_ NPs	Nano enzyme coupled with MIP as a bio-inspired body	Sulfadiazine	IC15: 0.08 mg and IC50 6.1 mg/L	[146]
AChE	DNA based materials	Electrochemical	Phytophthorapulmivora causing black pod rot in cacao pod	-	[147]
QD nanosensor	Gold nanoparticles	Immunosensor	Mycotoxins ZEA, DON, FB1/FB2	-	[148]
QD nanosensor	QDs	Fluorescence	Pathogens	-	[149]
Artificial nasal sensor	Carbon	Profile of volatile organic compounds	Pathogens depending on the organic compounds released	Sensitivity of 85% to 95%	[150]
Acetylcholinesterase on white paper using indophenol acetate	Enzyme	Colored antiphon	Paraoxon	3.5 μg/L	[151]
AChE	SMWCNTs	Electrochemical	Methylparathion, parathion and paraoxan	0.4 pM	[152]
Surface plasmon resonance (SPR)	MWCNTs	SPR	Cymbidium Mosaic virus	-	[153]
Nucleic acid nanosensor	CNTs	Immunosensor	Ganoderma boninse	0.2 ng/L	[154]
QD nanosensor	Quantum dots	Fluorescence	Pathogens and viruses	-	[155]

**Table 8:** Different types of nanobiosensors in environmental applications.

Nanobiosensor	Nanomaterials Used	Type of Sensor	Applications (Detection)	Limit of Detection (LOD)	Ref
AChE	SWCNTs and MWCNTs	Electrochemical	Parathion, Pesticides methylparathion, and paraoxan	0.5 pM	[161]
Plant hormone sensor	Receptor DAD2 from Petunia hybrid and HTLT from Striga hermohthica with green fluorescent protein	Fluorescent	Strigolactones as signaling molecules for plant growth and parasitism	High quantity testing approach	[162]
Smart	Zinc oxide and Copper	Electrochemical	Enhance the germination of tomato chili and cucurbits in Mexico	60 ppm	[163]
Acetylcholinesterase	Cholinergic enzyme	Amperometric	Chlorpyrifos	0.02 μg/L	[164]
Acetylcholinesterase immobilised on CuO	Cholinergic enzyme	Square wave voltammetry (SWV)	Malathion	0.33 ng/L	[165]
Acetylcholinesterase with MWCNTs	Enzyme	Differential pulse voltammetry	Paraoxon	2 ng/mL	[166]
Pesticide nanobiosensor	Graphene with molecularly imprinted polymers	Electrochemical	Pesticide revealing of chlorothlonil and chlorpyrifos methyl	0.14 mg/L	[167]
Molecular imprinted polymer	Mesoporous molecular sieves entrenched with carbon dots	Electrochemical	Natural plant Kaempferol polyphenol	14 μg/L	[168]
Agricultural nanosensor	ZnO and Cu	Electrochemical	Improve the germination of tomatoes, peppers, and other vegetables in Mexico	60 ppm	[169]

**Table 9:** Different types of nanobiosensors in the food industry.

Nanobiosensor	Nanomaterials Used	Type of Sensor	Applications (Detection)	Limit of Detection (LOD)	Ref
Toxin	SWCNTs	Optical and piezoelectric	Small toxins in food molecules	nM-fM	[173]
Aptamer sensor	Carbon	Aptamer	Antibiotic traces of carcinogens and acrylamide	12 ng/mL	[174]
Surface plasmon resonance	Carbon	Cantilever	Aflatoxins in rice and peanut	2.5 ppb	[175]
Melamine	DNA-Cu NPs	Fluorescence	Melamine in milk	50–100 µmol/L	[176]
Chemosynthetic mimotope peptide	Chemosynthetic peptide	Immunochromatographic	Ochratoxin A	0.196 ng/mL	[177]
Nanosensor	Sol-gel based ZnO NPs	*β*-galactosidase	E.coli	10^1^ CFU/mL	[178]
Array sensor	Carbon	Molecular array	Screening of genetically modified organism	-	[179]
Viscosity	Magnetic particles	Variation in viscosity	Pathogens	-	[180]
Ag NPs	Urease-antibody	ELISA	Salmonella in food samples	10^2^ CFU/mL	[181]
Antibody	ZnO	Sandwich ELISA	Salmonella typhimurium	-	[182]
Biofilm	Graphite oxide based aptamers	Electrochemical work station—DPV	Biofilms and Zearalenone Ochratoxin A	1.79 ng/mL–1.48 ng/mL	[183]
Carbon nanofibers (CNFs)	Nucleic acid aptamers	Electrochemical work station-SWV	Vibrio cholerae	1.25 × 10^−14^	[184]
ZIF-8 Gold nanoparticles/ Chitosan	Nucleic acid aptamers	Electrochemical work station—DPV	Bacillus cereus	3 CFU/mL	[185]
Multichannel nanosensor	Carbon particles	Immunosensor	Mainly used to measure the concentration of sweets such as saccharin, cyclamate, glucose, and sucrose	Saccharin 6–16 mM	[186]
Fe_3_O_4_ nanoparticles	Antibody	ELISA	Salmonella in milk	35 CFU/mL	[187]
Magnetic nanosensor	Antibody	ELISA with sandwich structure	Salmonella in chicken	10 CFU/mL	[188]
ZnO nanosensor	Antibody	ELISA with sandwich structure	Salmonella typhimurium	5 CFU/mL	[189]

## References

[1] Nehra A., Singh K.P. (2015). Current trends in nanomaterial embedded field effect transistor-based biosensor. Biosens. Bioelectron..

[2] Ong C.B., Ng L.Y., Mohammad A.W. (2018). A review of ZnO nanoparticles as solar photocatalysts: Synthesis, mechanisms and applications. Renew. Sustain. Energy Rev..

[3] Palanisamy P., Raichur A.M. (2009). Synthesis of spherical NiO nanoparticles through a novel biosurfactant mediated emulsion technique. Mater. Sci. Eng. C.

[4] Roy A., Ray A., Saha S., Ghosh M., Das T., Satpati B., Nandi M., Das S. (2018). NiO-CNT composite for high performance supercapacitor electrode and oxygen evolution reaction. Electrochim. Acta.

[5] Umapathi R., Raju C.V., Ghoreishian S.M., Rani G.M., Kumar K., Oh M.-H., Park J.P., Huh Y.S. (2022). Recent advances in the use of graphitic carbon nitride-based composites for the electrochemical detection of hazardous contaminants. Coord. Chem. Rev..

[6] Choi N., Park B., Lee M., Umapathi R., Oh S., Cho Y., Huh Y. (2021). Fabrication of Carbon Disulfide Added Colloidal Gold Colorimetric Sensor for the Rapid and On-Site Detection of Biogenic Amines. Sensors.

[7] Baird C.L., Myszka D.G. (2001). Current and emerging commercial optical biosensors. J. Mol. Recognit..

[8] Diculescu V.C., Chiorcea-Paquim A.M., Oliveira-Brett A.M. (2016). Applications of a DNA-electrochemical biosensor. TrAC Trends Anal. Chem..

[9] Muthamma K., Sunil D., Shetty P. (2021). Carbon dots as emerging luminophores in security inks for anti-counterfeit applications—An up-to-date review. Appl. Mater. Today.

[10] Kulkarni M.B., Goel S.G. (2020). Advances in continuous-flow based microfluidic PCR devices—A review. Eng. Res. Express.

[11] Umapathi R., Park B., Sonwal S., Rani G.M., Cho Y., Huh Y.S. (2022). Advances in optical-sensing strategies for the on-site detection of pesticides in agricultural foods. Trends Food Sci. Technol..

[12] Kulkarni M.B., Velmurugan K., Prasanth E., Amreen K., Nirmal J., Goel S. (2021). Smartphone enabled miniaturized temperature controller platform to synthesize nio/cuo nanoparticles for electrochemical sensing and nanomicelles for ocular drug delivery applications. Biomed. Microdevices.

[13] Kulkarni M.B., Us J., Amreen K., Goel S. (2021). Portable Thermal Management Platform for Synthesis of ZnO Nanoparticle in a Microfluidic Device: Validated for Electrochemical Sensing and Glucose Fuel Cell Applications. IEEE Trans. Electron Devices.

[14] Umapathi R., Ghoreishian S.M., Sonwal S., Rani G.M., Huh Y.S. (2021). Portable electrochemical sensing methodologies for on-site detection of pesticide residues in fruits and vegetables. Coord. Chem. Rev..

[15] PPatra J.K., Das G., Fraceto L.F., Campos E.V.R., del Pilar Rodriguez-Torres M., Acosta-Torres L.S., Diaz-Torres L.A., Grillo R., Swamy M.K., Sharma S. (2018). Nano based drug delivery systems: Recent developments and future prospects 10 Technology 1007 Nanotechnology 03 Chemical Sciences 0306 Physical Chemistry (incl. Structural) 03 Chemical Sciences 0303 Macromolecular and Materials Chemistry 11 Medical and He. J. Nanobiotechnol..

[16] Mashinchian O., Johari-Ahar M., Ghaemi B., Rashidi M., Barar J., Omidi Y. (2014). Impacts of quantum dots in molecular detection and bioimaging of cancer. BioImpacts.

[17] Umapathi R., Rani G.M., Kim E., Park S., Cho Y., Huh Y.S. (2022). Sowing kernels for food safety: Importance of rapid on-site detction of pesticide residues in agricultural foods. Food Front..

[18] Zhang Q., Uchaker E., Candelaria S.L., Cao G. (2013). Nanomaterials for energy conversion and storage. Chem. Soc. Rev..

[19] Aula S., Lakkireddy S., Jamil K., Kapley A., Swamy A.V.N., Lakkireddy H.R. (2015). Biophysical, biopharmaceutical and toxicological significance of biomedical nanoparticles. RSC Adv..

[20] Kulkarni M.B., Goel S. (2020). Microfluidic devices for synthesizing nanomaterials—A review. Nano Express.

[21] Umapathi R., Sonwal S., Lee M.J., Rani G.M., Lee E.-S., Jeon T.-J., Kang S.-M., Oh M.-H., Huh Y.S. (2021). Colorimetric based on-site sensing strategies for the rapid detection of pesticides in agricultural foods: New horizons, perspectives, and challenges. Coord. Chem. Rev..

[22] Kulkarni M.B., Ayachit N.H., Aminabhavi T.M. (2022). Biosensors and Microfluidic Biosensors: From Fabrication to Application. Biosensors.

[23] Puneeth S.B., Kulkarni M.B., Goel S.G. (2021). Microfluidic viscometers for biochemical and biomedical applications: A review. Eng. Res. Express.

[24] Kwon S.H., Lee S., Jang J., Seo Y., Lim H.Y. (2018). A point-of-care diagnostic system to influenza viruses using chip-based ultra-fast PCR. J. Med. Virol..

[25] Wang D., Chan H.N., Liu Z., Micheal S., Li L., Baniani D.B., Tan M.J.A., Huang L., Wang J. (2020). Recent Developments in Microfluidic-Based Point-of-care Testing (POCT) Diagnoses. Nanotechnol. Microfluid..

[26] Chen J., Meng H., Tian Y., Yang R., Du D., Li Z., Qu L., Lin Y. (2019). Recent advances in functionalized MnO 2 nanosheets for biosensing and biomedicine applications. Nanoscale Horiz..

[27] Jamkhande P.G., Ghule N.W., Bamer A.H., Kalaskar M.G. (2019). Metal nanoparticles synthesis: An overview on methods of preparation, advantages and disadvantages, and applications. J. Drug Deliv. Sci. Technol..

[28] Xu J., Hu Y., Wang S., Ma X., Guo J. (2020). Nanomaterials in electrochemical cytosensors. Analyst.

[29] Hao N., Nie Y., Zhang J.X.J. (2019). Microfluidics for silica biomaterials synthesis: Opportunities and challenges. Biomater. Sci..

[30] Ma M., Yang Y., Chen Y., Wu F., Li W., Lyu P., Ma Y., Tan W., Huang W. (2019). Synthesis of hollow flower-like FE_3_O_4_/MnO_2_/Mn_3_O_4_ magnetically separable microspheres with valence heterostructure for dye degradation. Catalysts.

[31] Vaidya S., Rastogi P., Agarwal S., Gupta S.K., Ahmad T., Antonelli J.A.M., Ramanujachary K.V., Lofland S.E., Ganguli A.K. (2008). Nanospheres, nanocubes, and nanorods of nickel oxalate: Control of shape and size by surfactant and solvent. J. Phys. Chem. C.

[32] Karthikeyan S., Suresh A., Sathyanadan M., Mahesh P. (2022). A competent top down approach methodology for design and development of commercial vehicle box type exhaust after treatment system. Mater. Today Proc..

[33] Olatomiwa A.L., Adam T., Gopinath S.C.B., Kolawole S.Y., Olayinka O.H., Hashim U. (2022). Graphene synthesis, fabrication, characterization based on bottom-up and top-down approaches: An overview. J. Semicond..

[34] Young C.W., Lien C.C., Ay C., Pan P.C. (2013). Computer simulation analysis of microchannel for a continuous-flow PCR chip. Appl. Mech. Mater..

[35] Sharma E., Rathi R., Misharwal J., Sinhmar B., Kumari S., Dalal J., Kumar A. (2022). Evolution in Lithography Techniques: Microlithography to Nanolithography. Nanomaterials.

[36] Zhou H., Jiang Y., Dmuchowski C.M., Ke C., Deng J. (2022). Electric-Field-Assisted Contact Mode Atomic Force Microscope-Based Nanolithography With Low Stiffness Conductive Probes. J. Micro Nano-Manuf..

[37] Shin H.W., Son J.Y. (2022). Ferroelectric domain wall current corresponding to ferroelectric domain structures of BaTiO_3_ nanodots fabricated by dip-pen nanolithography. Ceram. Int..

[38] Martínez L.M., Cruz-Angeles J., Vázquez-Dávila M., Martínez E., Cabada P., Navarrete-Bernal C., Cortez F. (2022). Mechanical Activation by Ball Milling as a Strategy to Prepare Highly Soluble Pharmaceutical Formulations in the Form of Co-Amorphous, Co-Crystals, or Polymorphs. Pharmaceutics.

[39] Valenti C., Federici M.I., Masciotti F., Marinucci L., Xhimitiku I., Cianetti S., Pagano S. (2022). Mechanical properties of 3D-printed prosthetic materials compared with milled and conventional processing: A systematic review and meta-analysis of in vitro studies. J. Prosthet. Dent..

[40] Hu Y., Liu T., Chen N., Feng C., Lu W., Guo H. (2022). Simultaneous bio-reduction of nitrate and Cr(VI) by mechanical milling activated corn straw. J. Hazard. Mater..

[41] Yang Y., Zhang Y., Yan M. (2022). A review on the preparation of thin-film YSZ electrolyte of SOFCs by magnetron sputtering technology. Sep. Purif. Technol..

[42] Sergievskaya A., Chauvin A., Konstantinidis S. (2022). Sputtering onto liquids: A critical review. Beilstein J. Nanotechnol..

[43] Arulkumar S., Parthiban S., Kwon J.Y., Uraoka Y., Bermundo J.P.S., Mukherjee A., Das B.C., Arulkumar S., Parthiban S., Kwon J. (2022). High mobility silicon indium oxide thin-film transistor fabrication by sputtering process. Vacuum.

[44] Liang Y.-J., Zhang L., Chen M., Fan L., Liao W., Xun Y., Fu L., Liu J., Liu F., Yang A. (2020). Anisotropic shaped Fe_3_O_4_ nanoparticles: Microwave-assisted thermal decomposition synthesis and their electromagnetic properties. AIP Adv..

[45] Lesnikovich A., Ivashkevich O., Levchik S., Balabanovich A., Gaponik P., Kulak A. (2002). Thermal decomposition of aminotetrazoles. Thermochim. Acta.

[46] Xu J., Zhou P., Zhang C., Yuan L., Xiao X., Dai L., Huo K. (2022). Striding the threshold of photocatalytic lignin-first biorefining via a bottom-up approach: From model compounds to realistic lignin. Green Chem..

[47] Binderbauer P.J., Kienberger T., Staubmann T. (2022). Synthetic load profile generation for production chains in energy intensive industrial subsectors via a bottom-up approach. J. Clean. Prod..

[48] Moden E.M., Plăiașu A.G. (2020). Advantages and Disadvantages of Chemical Methods in the Elaboration of Nanomaterials. Ann. “Dunarea de Jos” Univ. Galati. Fascicle IX Metall. Mater. Sci..

[49] Sansenya T., Masri N., Chankhanittha T., Senasu T., Piriyanon J., Mukdasai S., Nanan S. (2022). Hydrothermal synthesis of ZnO photocatalyst for detoxification of anionic azo dyes and antibiotic. J. Phys. Chem. Solids.

[50] Ahmadpour G., Nilforoushan M.R., Boroujeny B.S., Tayebi M., Jesmani S.M. (2022). Effect of substrate surface treatment on the hydrothermal synthesis of zinc oxide nanostructures. Ceram. Int..

[51] Chen Y., Hong Y., Ma Y., Li J. (2010). Synthesis and formation mechanism of urchin-like nano/micro-hybrid α-MnO_2_. J. Alloy. Compd..

[52] Ahmad S., Aadil M., Ejaz S.R., Akhtar M.U., Noor H., Haider S., Alsafari I.A., Yasmin G. (2022). Sol-gel synthesis of nanostructured ZnO/SrZnO_2_ with boosted antibacterial and photocatalytic activity. Ceram. Int..

[53] Zangeneh A., Vatani A., Fakhroeian Z., Peyghambarzadeh S. (2016). Experimental study of forced convection and subcooled flow boiling heat transfer in a vertical annulus using different novel functionalized ZnO nanoparticles. Appl. Therm. Eng..

[54] Song X., Díaz-Cuenca A. (2022). Sol–Gel Synthesis of Endodontic Cements: Post-Synthesis Treatment to Improve Setting Performance and Bioactivity. Materials.

[55] Zhao N., Wu Q., Zhang X., Yang T., Li D., Zhang X., Ma C., Liu R., Xin L., He M. (2022). Chemical vapor deposition growth of single-walled carbon nanotubes from plastic polymers. Carbon.

[56] Sun J., Han M., Gu Y., Yang Z.X., Zeng H. (2018). Recent Advances in Group III–V Nanowire Infrared Detectors. Adv. Opt. Mater..

[57] Hernandez Ruiz K., Wang Z., Ciprian M., Zhu M., Tu R., Zhang L., Luo W., Fan Y., Jiang W. (2022). Chemical Vapor Deposition Mediated Phase Engineering for 2D Transition Metal Dichalcogenides: Strategies and Applications. Small Sci..

[58] Yogalakshmi K.N., Poornima Devi T., Sivashanmugam P., Kavitha S., Yukesh Kannah R., Sunita V.S., Adish K., Gopalakrishnan K., Rajesh Banu J. (2022). Lignocellulosic biomass-based pyrolysis: A comprehensive review. Chemosphere.

[59] Al-Rumaihi A., Shahbaz M., Mckay G., Mackey H., Al-Ansari T. (2022). A review of pyrolysis technologies and feedstock: A blending approach for plastic and biomass towards optimum biochar yield. Renew. Sustain. Energy Rev..

[60] Wang X., Feng Y., Dong P., Huang J. (2019). A Mini Review on Carbon Quantum Dots: Preparation, Properties, and Electrocatalytic Application. Front. Chem..

[61] Racik M., Manikandan A., Mahendiran M., Madhavan J., Victor Antony Raj M., Mohamed M.G., Maiyalagan T. (2020). Hydrothermal synthesis and characterization studies of α-Fe_2_O_3_/MnO_2_ nanocomposites for energy storage supercapacitor application. Ceram. Int..

[62] Uhl A.M., Andrew J.S. (2020). Sol-Gel Based Electrospray Synthesis of Barium Titanate Nanoparticles. IEEE Trans. Nanobiosci..

[63] Rao C.N.R., Kulkarni G.U., Govindaraj A., Satishkumar B.C., Thomas P.J. (2000). Metal nanoparticles, nanowires, and carbon nanotubes. Pure Appl. Chem..

[64] Chaudhary J., Tailor G., Kumar D. (2020). Synthesis and d Characterizatio on of Chromium m nanoparticles by thermal decomposition method. Mater. Today Proc..

[65] Velmurugan K., Kulkarni M.B., Gupta I., Das R., Goel S., Nirmal J., Mohanan P.V. (2022). Role of Microfluidics in Drug Delivery. Microfluidics and Multi Organs on Chip.

[66] Kulkarni M.B., Goel S. (2021). A Review on Recent Advancements in Chamber-Based Microfluidic PCR Devices. Microelectron. Signal Process..

[67] Kulkarni M.B., Salve M., Goel S. (2021). Miniaturized Thermal Monitoring Module with CO_2_ Laser Ablated Microfluidic Device for Electrochemically Validated DNA Amplification. IEEE Trans. Instrum. Meas..

[68] Becker H., Dietz W. (1998). Microfluidic devices for μ -TAS applications fabricated by polymer hot embossing. Microfluid. Devices Syst..

[69] Prakash R., Kaler K.V.I.S. (2007). An integrated genetic analysis microfluidic platform with valves and a PCR chip reusability method to avoid contamination. Microfluid. Nanofluid..

[70] Gavrilaș S., Ursachi C., Perța-Crișan S., Munteanu F.-D. (2022). Recent Trends in Biosensors for Environmental Quality Monitoring. Sensors.

[71] Soldatkin O.O., Piliponskiy I.I., Rieznichenko L.S., Gruzina T.G., Dybkova S.M., Dzyadevych S.V., Soldatkin A.P. (2022). Application of gold nanoparticles for improvement of analytical characteristics of conductometric enzyme biosensors. Appl. Nanosci..

[72] Giepmans B.N.G., Adams S.R., Ellisman M.H., Tsien R.Y. (2006). The fluorescent toolbox for assessing protein location and function. Science.

[73] Tian Z., Ge X., Wang Y., Xu J. (2019). Nanoparticles and Nanocomposites with Microfluidic Technology.

[74] Naresh V., Lee N. (2021). A review on biosensors and recent development of nanostructured materials-enabled biosensors. Sensors.

[75] Umapathi R., Kumar K., Rani G.M., Venkatesu P. (2019). Influence of biological stimuli on the phase behaviour of a biomedical thermoresponsive polymer: A comparative investigation of hemeproteins. J. Colloid Interface Sci..

[76] Tomeh M.A., Zhao X. (2020). Recent Advances in Microfluidics for the Preparation of Drug and Gene Delivery Systems. Mol. Pharm..

[77] Mostafa M., Ebnalwaled K., Saied H.A., Roshdy R. (2018). Effect of laser beam on structural, optical, and electrical properties of BaTiO_3_ nanoparticles during sol-gel preparation. J. Korean Ceram. Soc..

[78] Tamayo J., Kosaka P.M., Ruz J.J., Paulo Á.S., Calleja M. (2013). Biosensors based on nanomechanical systems. Chem. Soc. Rev..

[79] Baluta S., Meloni F., Halicka K., Szyszka A., Zucca A., Pilo M.I., Cabaj J. (2022). Differential pulse voltammetry and chronoamperometry as analytical tools for epinephrine detection using a tyrosinase-based electrochemical biosensor. Rcs Adv..

[80] Nasseri B., Soleimani N., Rabiee N., Kalbasi A., Karimi M., Hamblin M.R. (2018). Point-of-care microfluidic devices for pathogen detection. Biosens. Bioelectron..

[81] Clark L.C., Lyons C. (1962). Electrode Systems for Continuous Monitoring in Cardiovascular Surgery. Ann. N. Y. Acad. Sci..

[82] Das T.K., Das N.C. (2022). Advances on catalytic reduction of 4-nitrophenol by nanostructured materials as benchmark reaction. Int. Nano Lett..

[83] Tomar A.S., Gupta R., Bijanu A., Arya R., Mishra D., Singh A., Salammal S.T. (2022). Progress in fabrication and manufacturing of sodium aluminosilicate materials (geopolymers) as protective coating materials: A review. J. Polym. Res..

[84] Hecht H., Schomburg D., Kalisz H., Schmid R. (1993). The 3D structure of glucose oxidase from Aspergillus niger. Implications for the use of GOD as a biosensor enzyme. Biosens. Bioelectron..

[85] Feynman R.P., Leighton R.B., Sands M., Hafner E.M. (1965). The Feynman Lectures on Physics; Vol. I. Am. J. Phys..

[86] Fang H., Yang Z., Ong C., Li Y., Wang C. (1998). Preparation and magnetic properties of (Zn–Sn) substituted barium hexaferrite nanoparticles for magnetic recording. J. Magn. Magn. Mater..

[87] Murphy B.E.P., Grigg E.R.N. (1979). Radioassays. Semin. Nucl. Med..

[88] Willner I., Katz E., Willner B. (1997). Electrical contact of redox enzyme layers associated with electrodes: Routes to amperometric biosensors. Electroanalysis.

[89] Frew J.E., Hill H.A.O., Thomas J.D.R. (1987). Electron-transfer biosensors. Philos. Trans. R. Soc. B Biol. Sci..

[90] Turner A. (1985). Diabetes mellitus: Biosensors for research and management. Biosensors.

[91] Mukhopadhayay S. (2012). Immunodiagnosis as an aid for early detection of Fasciola gigantica by glutathione S-transferase (GST). J. Parasit. Dis..

[92] Ceylan A., Rumaiz A.K., Shah S.I. (2007). Inert gas condensation of evaporated Ni and laser ablated CoO. J. Appl. Phys..

[93] Arlinghaus H.F., Kwoka M.N., Jacobson K.B. (1997). Analysis of Biosensor Chips for Identification of Nucleic Acids. Anal. Chem..

[94] Drexler K.E. (2001). Machine-phase nanotechnology. Sci. Am..

[95] Cinel N.A., Bütün S., Özbay E. (2012). Electron beam lithography designed silver nano-disks used as label free nano-biosensors based on localized surface plasmon resonance. Opt. Express.

[96] Yao T., Rechnitz G. (1987). Amperometric enzyme-immunosensor based on ferrocene-mediated amplification. Biosensors.

[97] Martin C.R., Kohli P. (2003). The emerging field of nanotube biotechnology. Nat. Rev. Drug Discov..

[98] Tothill I.E. (2001). Biosensors developments and potential applications in the agricultural diagnosis sector. Comput. Electron. Agric..

[99] Bartlett P.N., Bradford V.Q., Albery W.J., Burns D.T., Miller J.N., Townshend A., Townshend A. (1990). The use of redox mediator modified glucose oxidase in amperometric enzyme electrodes. Philos. Trans. R. Soc. Lond. Ser. A Phys. Eng. Sci..

[100] Erickson K., Wilding P. (1993). Evaluation of a novel point-of-care system, the i-STAT portable clinical analyzer. Clin. Chem..

[101] Arora N. (2019). Recent Advances in Biosensors Technology. A Review. Octa J. Biosci..

[102] Ngounou B., Neugebauer S., Frodl A., Reiter S., Schuhmann W. (2004). Combinatorial synthesis of a library of acrylic acid-based polymers and their evaluation as immobilisation matrix for amperometric biosensors. Electrochim. Acta.

[103] Haniff M., Vajravelu A., Muhammad T., Mohamed A.G. (2021). Fundamentals of Biosensor—An Introduction with its Applications in the Engineering Perspective. Int. J. Adv. Eng. Manag..

[104] Kulkarni M.B., Enaganti P.K., Amreen K., Goel S. (2021). Integrated Temperature Controlling Platform to Synthesize ZnO Nanoparticles and its Deposition on Al-Foil for Biosensing. IEEE Sens. J..

[105] Vigneshvar S., Sudhakumari C.C., Senthilkumaran B., Prakash H. (2016). Recent advances in biosensor technology for potential applications—An overview. Front. Bioeng. Biotechnol..

[106] Mehrotra P. (2016). Biosensors and their applications—A review. J. Oral Biol. Craniofacial Res..

[107] Chen Y.T., Lee Y.C., Lai Y.H., Lim J.C., Huang N.T., Lin C.T., Huang J.J. (2020). Review of Integrated Optical Biosensors for Point-Of-Care Applications. Biosensors.

[108] Su H., Li S., Jin Y., Xian Z., Yang D., Zhou W., Mangaran F., Leung F., Sithamparanathan G., Kerman K. (2017). Nanomaterial-based biosensors for biological detections. Adv. Health Care Technol..

[109] Jain U., Saxena K., Hooda V., Balayan S., Singh A.P., Tikadar M., Chauhan N. (2021). Emerging vistas on pesticides detection based on electrochemical biosensors—An update. Food Chem..

[110] Kaur B., Kumar S., Kaushik B.K. (2022). Recent advancements in optical biosensors for cancer detection. Biosens. Bioelectron..

[111] Chadha U., Bhardwaj P., Agarwal R., Rawat P., Agarwal R., Gupta I., Panjwani M., Singh S., Ahuja C., Selvaraj S.K. (2022). Recent progress and growth in biosensors technology: A critical review. J. Ind. Eng. Chem..

[112] Miller C.A., Ho J.M.L., Bennett M.R. (2022). Strategies for Improving Small-Molecule Biosensors in Bacteria. Biosensors.

[113] Chu H., Wei L., Cui R., Wang J., Li Y. (2010). Carbon nanotubes combined with inorganic nanomaterials: Preparations and applications. Coord. Chem. Rev..

[114] Guan J., Sagar L.K., Li R., Wang D., Bappi G., Wang W., Watkins N., Bourgeois M.R., Levina L., Fan F. (2020). Quantum Dot-Plasmon Lasing with Controlled Polarization Patterns. ACS Nano.

[115] Jiang X., Herricks A.T., Xia Y. (2002). CuO Nanowires Can Be Synthesized by Heating Copper Substrates in Air. Nano Lett..

[116] Sonker R.K., Yadav B., Gupta V., Tomar M. (2019). Synthesis of CdS nanoparticle by sol-gel method as low temperature NO_2_ sensor. Mater. Chem. Phys..

[117] Krahne R., Morello G., Figuerola A., George C., Deka S., Manna L. (2011). Physical properties of elongated inorganic nanoparticles. Phys. Rep..

[118] Ouyang D., Huang Z., Choy W.C.H. (2018). Solution-Processed Metal Oxide Nanocrystals as Carrier Transport Layers in Organic and Perovskite Solar Cells. Adv. Funct. Mater..

[119] Sakdaphetsiri K., Teanphonkrang S., Schulte A. (2022). Cheap and Sustainable Biosensor Fabrication by Enzyme Immobilization in Commercial Polyacrylic Acid/Carbon Nanotube Films. ACS Omega.

[120] Chuong J.J.C.C., Rahman M., Ibrahim N., Heng L.Y., Tan L.L., Ahmad A. (2022). Harmful Microalgae Detection: Biosensors versus Some Conventional Methods. Sensors.

[121] Kulkarni M.B., Goyal S., Dhar A., Sriram D., Goel S. (2021). Miniaturized and IoT Enabled Continuous-Flow-Based Microfluidic PCR Device for DNA Amplification. IEEE Trans. Nanobiosci..

[122] Hashimoto M., Barany F., Soper S.A. (2006). Polymerase chain reaction/ligase detection reaction/hybridization assays using flow-through microfluidic devices for the detection of low-abundant DNA point mutations. Biosens. Bioelectron..

[123] Zhang C., Xu J., Wang J., Wang H. (2007). Continuous-flow polymerase chain reaction microfluidics by using spiral capillary channel embedded on copper. Anal. Lett..

[124] Lee D.S., Park S.H., Chung K.H., Pyo H.B. (2008). A disposable plastic-silicon micro PCR chip using flexible printed circuit board protocols and its application to genomic DNA amplification. IEEE Sens. J..

[125] Ren K., Zhou J., Wu H. (2013). Materials for microfluidic chip fabrication. Acc. Chem. Res..

[126] Mohan J.M., Amreen K., Kulkarni M.B., Javed A., Dubey S.K., Goel S. (2021). Optimized Ink Jetted Paper Device for Electroanalytical Detection of Picric Acid. Colloids Surf. B Biointerfaces.

[127] Jayapiriya U.S., Goel S. Optimization of carbon cloth bioelectrodes for enzyme-based biofuel cell for wearable bioelectronics. Proceedings of the 2020 IEEE 20th International Conference on Nanotechnology.

[128] Xuan X., Hossain F., Park J.Y. (2016). A Fully Integrated and Miniaturized Heavy-metal-detection Sensor Based on Micro-patterned Reduced Graphene Oxide. Sci. Rep..

[129] Kulkarni M.B., Goel S. (2021). Miniaturized DNA amplification platform with soft-lithographically fabricated continuous-flow PCR microfluidic device on a portable temperature controller. Microfluid. Nanofluid..

[130] Ali S., Hassan A., Hassan G., Eun C.H., Bae J., Lee C.H., Kim I.J. (2018). Disposable all-printed electronic biosensor for instantaneous detection and classification of pathogens. Sci. Rep..

[131] Tsaloglou M.N., Nemiroski A., Camci-Unal G., Christodouleas D.C., Murray L.P., Connelly J.T., Whitesides G.M. (2018). Handheld isothermal amplification and electrochemical detection of DNA in resource-limited settings. Anal. Biochem..

[132] Avargani V.M., Norton B., Rahimi A., Karimi H. (2021). Integrating paraffin phase change material in the storage tank of a solar water heater to maintain a consistent hot water output temperature. Sustain. Energy Technol. Assess..

[133] Park J., Park H. (2017). Thermal cycling characteristics of a 3D-printed serpentine microchannel for DNA amplification by polymerase chain reaction. Sens. Actuators A Phys..

[134] Sun Y., Satyanarayan M., Nguyen N.-T., Kwok Y.C. (2008). Continuous flow polymerase chain reaction using a hybrid PMMA-PC microchip with improved heat tolerance. Sens. Actuators B Chem..

[135] Trinh K.T.L., Lee N.Y. (2018). Glass-polytetrafluoroethylene-glass based sandwich microdevice for continuous-flow polymerase chain reaction and its application for fast identification of foodborne pathogens. Talanta.

[136] Kim P., Kwon K.W., Park M.C., Lee S.H., Kim S.M., Suh K.Y. (2008). Soft lithography for microfluidics: A Review. Biochip J..

[137] Whulanza Y., Aditya R., Arvialido R., Utomo M.S., Bachtiar B.M. (2017). Ease fabrication of PCR modular chip for portable DNA detection kit. AIP Conf. Proc..

[138] Zhang C., Xu J., Ma W., Zheng W. (2006). PCR microfluidic devices for DNA amplification. Biotechnol. Adv..

[139] Pal A., Kulkarni M.B., Gupta H., Ponnalagu R., Dubey S.K., Goel S. (2021). Portable and Autonomous Device for Real-time Colorimetric Detection: Validation for Phosphorous and Nitrite Detection. Sens. Actuators A Phys..

[140] Bhaiyya M., Kulkarni M.B., Pattnaik P.K., Goel S. (2021). Internet of things-enabled photomultiplier tube- and smartphone-based electrochemiluminescence platform to detect choline and dopamine using 3D-printed closed bipolar electrodes. Luminescence.

[141] Kulkarni M.B., Goel S. (2022). Recent advancements in integrated microthermofluidic systems for biochemical and biomedical applications—A review. Sens. Actuators A Phys..

[142] Soni A., Surana R.K., Jha S.K. (2018). Chemical Smartphone based optical biosensor for the detection of urea in saliva. Sens. Actuators B Chem..

[143] Yang Q., Li N., Li Q., Chen S., Wang H.L., Yang H. (2019). Amperometric sarcosine biosensor based on hollow magnetic Pt–Fe_3_O_4_@C nanospheres. Anal. Chim. Acta.

[144] Yerlikaya S., Broger T., MacLean E., Pai M., Denkinger C.M. (2017). A tuberculosis biomarker database: The key to novel TB diagnostics. Int. J. Infect. Dis..

[145] Sposito A.J., Kurdekar A., Zhao J., Hewlett I. (2018). Application of nanotechnology in biosensors for enhancing pathogen detection. Wiley Interdiscip. Rev. Nanomed. Nanobiotechnol..

[146] Ankri S., Mirelman D. (1999). Antimicrobial properties of allicin from garlic. Microbes Infect..

[147] D’Orazio P. (2003). Biosensors in clinical chemistry. Clin. Chim. Acta.

[148] Kulakovich O., Strekal N., Yaroshevich A., Maskevich S., Gaponenko S., Nabiev I., Woggon U., Artemyev M. (2002). Enhanced Luminescence of CdSe Quantum Dots on Gold Colloids. Nano Lett..

[149] Zhao X., Li M., Liu Y. (2019). Microfluidic-Based Approaches for Foodborne Pathogen Detection. Microorganisms.

[150] Kim S., Brady J., Al-Badani F., Yu S., Hart J., Jung S., Tran T.T., Myung N.V. (2021). Nanoengineering Approaches Toward Artificial Nose. Front. Chem..

[151] Sela M.N. (2001). Role of Treponema denticola in periodontal diseases. Crit. Rev. Oral Biol. Med..

[152] Wong Y., Zysman-Colman E. (2017). Purely Organic Thermally Activated Delayed Fluorescence Materials for Organic Light-Emitting Diodes. Adv. Mater..

[153] Antonacci A., Arduini F., Moscone D., Palleschi G., Scognamiglio V. (2018). Nanostructured (Bio)sensors for smart agriculture. TrAC Trends Anal. Chem..

[154] Taylor P., Safarpour H., Rad F., Basirat M., Shahryari F. (2012). Development of a quantum dots FRET-based biosensor for efficient detection of Polymyxa betae Development of a quantum dots FRET-based biosensor for efficient detection of *Polymyxa betae*. Can. J. Plant Pathol..

[155] Efros A.L., Delehanty J.B., Huston A.L., Medintz I.L., Barbic M., Harris T.D. (2018). Evaluating the potential of using quantum dots for monitoring electrical signals in neurons. Nat. Nanotechnol..

[156] Hu S., Jie Y., Jin K., Zhang Y., Guo T., Huang Q., Mei Q., Ma F., Ma H. (2022). All-in-One Digital Microfluidics System for Molecular Diagnosis with Loop-Mediated Isothermal Amplification. Biosensors.

[157] Garcia-Rey S., Nielsen J.B., Nordin G.P., Woolley A.T., Basabe-Desmonts L., Benito-Lopez F. (2022). High-Resolution 3D Printing Fabrication of a Microfluidic Platform for Blood Plasma Separation. Polymers.

[158] Tian J., Li Y., Dong J., Huang M., Lu J. (2018). Photoelectrochemical TiO_2_ nanotube arrays biosensor for asulam determination based on in-situ generation of quantum dots. Biosens. Bioelectron..

[159] Chen J., Fan L., Yang C., Wang S., Zhang M., Xu J., Luo S. (2020). Facile synthesis of Ag nanoparticles-loaded chitosan antibacterial nanocomposite and its application in polypropylene. Int. J. Biol. Macromol..

[160] Ndolomingo M.J., Bingwa N., Meijboom R. (2020). Review of supported metal nanoparticles: Synthesis methodologies, advantages and application as catalysts. J. Mater. Sci..

[161] Pundir C.S., Chauhan N. (2012). Acetylcholinesterase inhibition-based biosensors for pesticide determination: A review. Anal. Biochem..

[162] Ariffin S.A., Adam T., Hashim U., Sfaridah S.F., Zamri I., Uda M.N.A. (2013). Plant diseases detection using nanowire as biosensor transducer. Adv. Mater. Res..

[163] Sasirekha C., Arumugam S., Muralidharan G. (2018). Green synthesis of ZnO/carbon (ZnO/C) as an electrode material for symmetric supercapacitor devices. Appl. Surf. Sci..

[164] Teng H., Zuo Y., Jin Z., Wu Y., Yang Y. (2022). Associations between acetylcholinesterase-1 mutations and chlorpyrifos resistance in beet armyworm, Spodoptera exigua. Pestic. Biochem. Physiol..

[165] Bilal S., Nasir M., Hassan M.M., Rehman M.F.U., Sami A.J., Hayat A. (2022). A novel construct of an electrochemical acetylcholinesterase biosensor for the investigation of malathion sensitivity to three different insect species using a NiCr_2_O_4_/g-C_3_N_4_ composite integrated pencil graphite electrode. RSC Adv..

[166] Celik H., Soylemez S. (2022). An Electrochemical Acetylcholinesterase Biosensor Based on Fluorene(bisthiophene) Comprising Polymer for Paraoxon Detection. Electroanalysis.

[167] Temkin A.M., Uche U.I., Evans S., Anderson K.M., Perrone-Gray S., Campbell C., Naidenko O.V. (2022). Racial and social disparities in Ventura County, California related to agricultural pesticide applications and toxicity. Sci. Total Environ..

[168] He J., Zhang L., Xu L., Kong F., Xu Z. (2020). Development of Nanozyme-Labeled Biomimetic Immunoassay for Determination of Sulfadiazine Residue in Foods. Adv. Polym. Technol..

[169] Negrete J.C. (2020). Bioinformatics Nanotechnology an Option in Mexican Agriculture. J. Biotechnol. Bioin-Form. Res..

[170] Zarei M. (2017). Advances in point-of-care technologies for molecular diagnostics. Biosens. Bioelectron..

[171] Wang X., Gartia M.R., Jiang J., Chang T.W., Qian J., Liu Y., Liu X., Liu G.L. (2014). Audio jack based miniaturized mobile phone electrochemical sensing platform. Sens. Actuators B Chem..

[172] Li S.J., Xia N., Lv X.L., Zhao M.M., Yuan B.Q., Pang H. (2014). A facile one-step electrochemical synthesis of graphene/NiO nanocomposites as efficient electrocatalyst for glucose and methanol. Sens. Actuators B Chem..

[173] Gui Q., Lawson T., Shan S., Yan L., Liu Y. (2017). The application of whole cell-based biosensors for use in environmental analysis and in medical diagnostics. Sensors.

[174] Liss M., Petersen B., Wolf H., Prohaska E. (2002). An aptamer-based quartz crystal protein biosensor. Anal. Chem..

[175] Kaur B., Kumar S., Kaushik B.K. (2022). MXenes-Based Fiber-Optic SPR Sensor for Colorectal Cancer Diagnosis. IEEE Sens. J..

[176] El-Kemary M., Nagy N., El-Mehasseb I. (2013). Nickel oxide nanoparticles: Synthesis and spectral studies of interactions with glucose. Mater. Sci. Semicond. Process..

[177] Rhouati A., Hayat A., Hernandez D.B., Meraihi Z., Munoz R., Marty J.L. (2013). Development of an automated flow-based electrochemical aptasensor for on-line detection of Ochratoxin A. Sens. Actuators B Chem..

[178] Pandey P., Merwyn S., Agarwal G.S., Tripathi B.K., Pant S.C. (2012). Electrochemical synthesis of multi-armed CuO nanoparticles and their remarkable bactericidal potential against waterborne bacteria. J. Nanopart. Res..

[179] Juan-Colás J., Parkin A., Dunn K.E., Scullion M.G., Krauss T.F., Johnson S.D. (2016). The electrophotonic silicon biosensor. Nat. Commun..

[180] Puneeth S.B., Puranam S.A., Goel S. (2018). 3-D Printed Integrated and Automated Electro-Microfluidic Viscometer for Biochemical Applications. IEEE Trans. Instrum. Meas..

[181] Chou J.C., Wu C.Y., Kuo P.Y., Lai C.H., Nien Y.H., Wu Y.X., Lin S.H., Liao Y.H. (2019). The Flexible Urea Biosensor Using Magnetic Nanoparticles. IEEE Trans. Nanotechnol..

[182] Han S.-M., Kim Y.-W., Chun J.-H., Oh H.-B., Paek S.-H. (2018). Performance Characterization of Two-Dimensional Paper Chromatography-based Biosensors for Biodefense, Exemplified by Detection of Bacillus anthracis Spores. Biochip J..

[183] Wang L., Huo X., Qi W., Xia Z., Li Y., Lin J. (2020). Rapid and sensitive detection of Salmonella Typhimurium using nickel nanowire bridge for electrochemical impedance amplification. Talanta.

[184] Mofokeng T.P., Tetana Z.N., Ozoemena K.I. (2020). Defective 3D nitrogen-doped carbon nanotube-carbon fibre networks for high-performance supercapacitor: Transformative role of nitrogen-doping from surface-confined to diffusive kinetics. Carbon.

[185] Zhang P., Sun T., Rong S., Zeng D., Yu H., Zhang Z., Chang D., Pan H. (2019). A sensitive amperometric AChE-biosensor for organophosphate pesticides detection based on conjugated polymer and Ag-rGO-NH_2_ nanocomposite. Bioelectrochemistry.

[186] Zhang F., Zhang Q., Zhang D., Lu Y., Liu Q., Wang P. (2014). Biosensor analysis of natural and artificial sweeteners in intact taste epithelium. Biosens. Bioelectron..

[187] Cheng N., Zhu C., Wang Y., Du D., Zhu M.-J., Luo Y., Xu W., Lin Y. (2019). Nanozyme Enhanced Colorimetric Immunoassay for Naked-Eye Detection of Salmonella Enteritidis. J. Anal. Test..

[188] Zheng L., Cai G., Qi W., Wang S., Wang M.-H., Lin J. (2019). Optical Biosensor for Rapid Detection of Salmonella typhimurium Based on Porous Gold@Platinum Nanocatalysts and a 3D Fluidic Chip. ACS Sens..

[189] Huang F., Guo R., Xue L., Cai G., Wang S., Li Y., Liao M., Wang M., Lin J. (2020). An Acid-Responsive Microfluidic Salmonella Biosensor Using Curcumin as Signal Reporter and ZnO-Capped Mesoporous Silica Nanoparticles for Signal Amplification. Sens. Actuators B Chem..

[190] Hassan R.Y.A. (2022). Advances in Electrochemical Nano-Biosensors for Biomedical and Environmental Applications: From Current Work to Future Perspectives. Sensors.

[191] Maheswaran R., Shanmugavel B.P. (2022). A Critical Review of the Role of Carbon Nanotubes in the Progress of Next-Generation Electronic Applications. J. Electron. Mater..

[192] Karmakar P., Sahu P.K. (2022). Study and Analysis of Dielectrically Modulated Vertical Tunnel FET Biosensor Considering Non-Ideal Hybridization. Silicon.

[193] Pan Y., Qin Z., Kheiri S., Ying B., Pan P., Peng R., Liu X. (2021). Optical Printing of Conductive Silver on Ultrasmooth Nanocellulose Paper for Flexible Electronics. Adv. Eng. Mater..

[194] Dadras-Toussi O., Khorrami M., Titus A.S.C.L.S., Majd S., Mohan C., Abidian M.R. (2022). Multiphoton Lithography of Organic Semiconductor Devices for 3D Printing of Flexible Electronic Circuits, Biosensors, and Bioelectronics. Adv. Mater..

[195] Thakur M., Wang B., Verma M.L. (2022). Development and applications of nanobiosensors for sustainable agricultural and food industries: Recent developments, challenges and perspectives. Environ. Technol. Innov..

[196] Zhang H., Wang X., Wang J., Chen Q., Huang H., Huang L., Cao S., Ma X. (2020). UV–visible diffuse reflectance spectroscopy used in analysis of lignocellulosic biomass material. Wood Sci. Technol..

[197] Si H., Xu G., Jing F., Sun P., Zhao D., Wu D. (2020). A multi-volume microfluidic device with no reagent loss for low-cost digital PCR application. Sens. Actuators B Chem..

[198] Holzinger M., Le Goff A., Cosnier S. (2014). Nanomaterials for biosensing applications: A review. Front. Chem..

[199] Zhang X., Guo Q., Cui D. (2009). Recent advances in nanotechnology applied to biosensors. Sensors.

[200] Banigo A., Azeez T., Ejeta K., Lateef A., Ajuogu E. (2020). Nanobiosensors: Applications in biomedical technology. IOP Conf. Ser. Mater. Sci. Eng..

[201] Dewan N., Ahmed P., Chowdhury G., Pandit S., Dasgupta D. (2016). Nanotechnology based biosensors and its application. Pharma Innov. J..

[202] Tang J., Ibrahim M., Chakrabarty K., Karri R. (2018). Toward Secure and Trustworthy Cyberphysical Microfluidic Biochips. IEEE Trans. Comput. Des. Integr. Circuits Syst..

[203] Annabestani M., Shaegh A.M., Esmaeili-Dokht P., Fardmanesh M. An Intelligent Machine Learning-Based Sheath-free Microfluidic Impedance Flow cytometer. Proceedings of the 2020 10th International Conference on Computer and Knowledge Engineering (ICCKE).

[204] Koo S.-B., Jo D.-G., Park C.-Y., Kim Y.-S., Song H.-J., Kim J.-D. (2019). Low-cost miniaturization of gel document system using blue LED. Sens. Mater..

[205] Huang X., Duan Y., Zhao L., Liu S., Qin D., Zhang F., Lin D. (2020). Dexamethasone pharmacokinetics characteristics via sub-tenon microfluidic system in uveitis rabbits. J. Drug Deliv. Sci. Technol..

[206] Kant K., Shahbazi M.A., Dave V.P., Ngo T.A., Chidambara V.A., Than L.Q., Bang D.D., Wolff A. (2018). Microfluidic devices for sample preparation and rapid detection of foodborne pathogens. Biotechnol. Adv..

[207] Shetti N.P., Bukkitgar S.D., Reddy K.R., Reddy C.V., Aminabhavi T.M. (2019). ZnO-based nanostructured electrodes for electrochemical sensors and biosensors in biomedical applications. Biosens. Bioelectron..

[208] Soy S., Sharma S.R., Nigam V.K. (2022). Bio-fabrication of thermozyme-based nano-biosensors: Their components and present scenario. J. Mater. Sci. Mater. Electron..

[209] Singh S., Numan A., Cinti S. (2022). Electrochemical nano biosensors for the detection of extracellular vesicles exosomes: From the benchtop to everywhere?. Biosens. Bioelectron..

[210] Kulkarni M.B., Yashas, Enaganti P.K., Amreen K., Goel S. (2020). Internet of Things enabled portable thermal management system with microfluidic platform to synthesize MnO_2_ nanoparticles for electrochemical sensing. Nanotechnology.

